# Fibulin-3 is necessary to prevent cardiac rupture following myocardial infarction

**DOI:** 10.1038/s41598-023-41894-9

**Published:** 2023-09-11

**Authors:** Lucy A. Murtha, Sean A. Hardy, Nishani S. Mabotuwana, Mark J. Bigland, Taleah Bailey, Kalyan Raguram, Saifei Liu, Doan T. Ngo, Aaron L. Sverdlov, Tamara Tomin, Ruth Birner-Gruenberger, Robert D. Hume, Siiri E. Iismaa, David T. Humphreys, Ralph Patrick, James J. H. Chong, Randall J. Lee, Richard P. Harvey, Robert M. Graham, Peter P. Rainer, Andrew J. Boyle

**Affiliations:** 1 Faculty of Health and Medicine, The University of Newcastle, Newcastle, NSW Australia; 2 Hunter Medical Research Institute, Newcastle, NSW 2305 Australia; 3 Department of Cardiology and Clinical Pharmacology, Basil Hetzel Institute, The University of Adelaide, The Queen Elizabeth Hospital, Adelaide, SA Australia; 4 Department of Cardiovascular Medicine, John Hunter Hospital, Newcastle, NSW Australia; 5 Institute of Chemical Technologies and Analytics, Faculty of Technical Chemistry, Technische Universität Wien, Vienna, Austria; 6 Diagnostic and Research Institute of Pathology, Medical University of Graz, Graz, Austria; 7 Centre for Heart Research, Westmead Institute for Medical Research, The University of Sydney, Sydney, NSW Australia; 8 Victor Chang Cardiac Research Institute, Sydney, NSW Australia; 9 St Vincent’s Clinical School, UNSW, Sydney, Kensington, NSW Australia; 10 Department of Cardiology, Westmead Hospital, Sydney, NSW Australia; 11 Faculty of Medicine and Health, The University of Sydney, Sydney, NSW Australia; 12 Department of Medicine, Division of Cardiology, University of California San Francisco, San Francisco, CA USA; 13 Edyth and Eli Broad Center for Regenerative Medicine and Stem Cell Research, University of California San Francisco, San Francisco, CA USA; 14 Cardiovascular Research Institute, University of California San Francisco, San Francisco, CA USA; 15 School of Biotechnology and Molecular Bioscience, UNSW, Sydney, Kensington, NSW Australia; 16 Division of Cardiology, Medical University of Graz, Graz, Austria; 17 BioTechMed Graz, Graz, Austria

**Keywords:** Cardiovascular biology, Preclinical research, Cardiovascular diseases

## Abstract

Despite the high prevalence of heart failure in the western world, there are few effective treatments. Fibulin-3 is a protein involved in extracellular matrix (ECM) structural integrity, however its role in the heart is unknown. We have demonstrated, using single cell RNA-seq, that fibulin-3 was highly expressed in quiescent murine cardiac fibroblasts, with expression highest prior to injury and late post-infarct (from ~ day-28 to week-8). In humans, fibulin-3 was upregulated in left ventricular tissue and plasma of heart failure patients. Fibulin-3 knockout (*Efemp1*^−/−^) and wildtype mice were subjected to experimental myocardial infarction. Fibulin-3 deletion resulted in significantly higher rate of cardiac rupture days 3–6 post-infarct, indicating a weak and poorly formed scar, with severe ventricular remodelling in surviving mice at day-28 post-infarct. Fibulin-3 knockout mice demonstrated less collagen deposition at day-3 post-infarct, with abnormal collagen fibre-alignment. RNA-seq on day-3 infarct tissue revealed upregulation of ECM degradation and inflammatory genes, but downregulation of ECM assembly/structure/organisation genes in fibulin-3 knockout mice. GSEA pathway analysis showed enrichment of inflammatory pathways and a depletion of ECM organisation pathways. Fibulin-3 originates from cardiac fibroblasts, is upregulated in human heart failure, and is necessary for correct ECM organisation/structural integrity of fibrotic tissue to prevent cardiac rupture post-infarct.

## Introduction

Fibrosis of the myocardium is the hallmark of post-infarction heart failure^1–4^. The fundamental mechanisms that lead to cardiac fibrosis following myocardial infarction (MI) are incompletely understood. Therapies to prevent or reverse cardiac fibrosis are, at best, partially effective, and new therapies are needed. A deeper understanding of the different cellular and molecular processes causing cardiac fibrosis is required for the effective design of new medical therapies for heart failure.

Fibulin-3 is an ECM glycoprotein encoded by the *Efemp1* (epidermal growth factor containing fibulin extracellular matrix protein 1) gene located on chromosome 2p16 (human)^5^. Fibulin-3 is highly expressed by epithelial and endothelial cells and localises to basement membranes^5^, but was first identified in senescent and quiescent fibroblasts^6^. It is a member of the fibulin family of proteins that are characterised by tandem arrays of calcium binding EGF domains and a C-terminal fibulin-type module. Human fibulin-3 is a 493-amino acid protein with a molecular mass of 55 kDa. The biological function of fibulin-3 remains incompletely understood, however it appears to be involved in ECM structural integrity^7,8^. The fibulin-3 protein is expressed in many human tissues including the heart^9^. Despite literature characterising various effects of fibulin-3 knockout or mutation^7,10,11^, the role of fibulin-3 in the heart, particularly its role in heart failure, is currently unknown. Therefore, we aimed to characterise fibulin-3 in cardiac fibrosis and hypothesised that fibulin-3 plays a fundamental role in scar tissue structural integrity after myocardial infarction (MI).

## Materials and methods

### Ethics statement

All experiments involving animals were performed on male and female *Efemp1*^−/−^ and wildtype (WT; *Efemp1*^+*/*+^) littermate mice aged 3–4 months generated from heterozygous *Efemp1*^+*/-*^ crosses (C57Bl/6J background; Australian BioResources), in accordance with the Australian Code for the Care and Use of Animals for Scientific Purposes. All animal experiments were approved by the Ethics Committee of the University of Newcastle. All experiments and analysis were performed in accordance with the ARRIVE guidelines by an investigator blinded to genotype. Animal experiments were randomised and no exclusion criteria were set following randomisation.

The use of human myocardial tissue samples was approved by the Ethics committee of the Medical University of Graz in accordance with the Declaration of Helsinki (28–508 ex 15/16). Left ventricular (LV) myocardial tissue samples were obtained from end-stage failing heart explants that were not accepted for transplantation. End-stage heart failure patients provided informed written consent. The use of blood samples was approved by the Ethics committee of the University of South Australia. After obtaining written informed consent, blood samples were obtained from acute heart failure patients and healthy volunteers. All protocols were conducted in accordance with the Australian National Statement on Ethical Conduct in Human Research 2007.

### Analysis of online single cell/nuclei and bulk RNA-seq datasets

An extended description of RNA-seq analysis is available in the Supplementary Methods online. Briefly, previously published single-cell RNA-seq and single-nuclei RNA-seq datasets were downloaded from ArrayExpress (accession codes E-MTAB-7376^12^ and E-MTAB-7869^13^). Bulk RNA-Seq datasets were downloaded from the gene expression omnibus (identifiers GSE141929^14^ and GSE114695^15^). Single cell RNA-seq and single nuclei RNA-seq datasets were analysed using the *Seurat* version 3.1.4 R package^16^. For bulk RNA-seq, raw Fastq files were processed using Trimmomatic^17^ and STAR aligner^18^, with differentially expressed (DE) genes evaluated using DESeq2^19^.

### Human tissue preparation and analysis

Left ventricular tissue specimens from end-stage failing and non-failing hearts were subjected to LC–MS/MS analysis. Details are described in the Supplementary Methods online. LC–MS/MS data was searched against SwissProt human proteins and common laboratory contaminants (FDR for identification < 1%) and label-free protein quantification were performed using MaxQuant (v1.6.1.0)^20^.

Blood samples were collected from healthy subjects aged ≥ 55 years with no prior history of cardiac dysfunction or hypertension and from patients with a primary diagnosis of acute heart failure. Plasma fibulin-3, N-terminal pro b-type natriuretic peptide (NT-proBNP) and high-sensitivity C-reactive protein (hs-CRP) concentrations were analysed using an ELISA kit (MyBioSource, MBS2022107), an immunoassay (Elecsys E 170, Roche Diagnostics) and a latex-enhanced immunoturbidometric assay (Olympus au5400), respectively.

### Murine surgical protocol and tissue analysis

Experimental MI was performed on *Efemp1*^−/−^ and WT littermate mice as previously described^21^. *Efemp1*^−/−^ mice were generated de novo in collaboration with Australian BioResources (Garvan Institute of Medical Resources). Briefly, mice were anaesthetised with isoflurane in oxygen and medical air (50:50%; 5% induction, 1.5–2% maintenance), intubated and mechanically ventilated. Mice underwent permanent left anterior descending artery (LAD) ligation and received a bolus of subcutaneous saline and the analgesic, carprofen (5 mg/kg). Transthoracic echocardiography was performed on all mice with a MS550D transducer (Vevo1100, Visualsonics, Toronto, Canada) under light isoflurane anaesthesia at baseline, and days 2 and 28 post-MI^22^. Additionally, the right lungs were investigated for lung congestion using ultrasound (MoLUS—Mouse Lung UltraSound) as outlined in Villalba-Orero et al. (2017)^22^. Briefly, the right pulmonary field was scanned longitudinally to visual the pleural line, space and layers. Lungs were classified and scored according to pre-defined parameters (MoLUS score). All scanning and analysis was performed blinded to genotype. Mice were euthanised at day 3 or 28 post-MI, via exsanguination under deep anaesthesia (isoflurane 5%), hearts and lungs were weighed (wet weight), and tibias measured.

Masson’s trichrome histological staining and collagen deposition analysis (circumferential extent) was performed on formalin-fixed hearts (3-day post-MI, n = 10/group; 28-day post-MI, n = 16 *Efemp1*^−/−^, n = 25 WT; male and female mice ~ equal distribution). qPCR analysis of collagen-I and -III and matrix metalloproteinase (Mmp)-2, -9 was performed on snap frozen infarct, border and remote zone tissue (3-day post-MI, n = 5/group, male and female mice ~ equal distribution); detailed methods and primers are described in the Supplementary Methods online. Second harmonic generation two-photon imaging was conducted on formalin fixed, CUBIC-cleared infarct zone tissue (3-day post-MI, n = 5–6/group, male mice). RNA-seq analysis was performed on snap-frozen infarct zone tissue (3-day post-MI, n = 5–7/group, male mice). Post RNA-seq analysis was performed using edgeR to identify differentially expressed genes^23^ (statistical significance assigned as FDR ≤ 0.05), and subsequent gene set enrichment analysis (GSEA) 4.0.3, Broad Institute, desktop application build-23, built on 21 Nov 2019. Detailed methods of GSEA are described in the Supplementary Methods online. Investigators were blinded to genotype when collecting the tissue to prevent unintended bias.

### Statistical analysis

Statistically significant differences between two groups were determined using the Mann–Whitney’s U test (non-parametric) or two-way Student’s t-test (parametric), and two-way ANOVA was used to compare between multiple groups, with Tukey’s multiple comparison testing. Survival was compared by Kaplan–Meier analysis (log-rank (Mantel-Cox) test), and linear regression analysis performed to determine relationship differences. *p* < 0.05 was accepted as significant. Data is displayed as mean ± standard deviation, or median (interquartile range—IQR) unless otherwise stated.

## Results

### Fibulin-3 expression occurs predominantly in cardiac fibroblasts in uninjured hearts and is dynamically altered post-MI in a time-dependent manner

**Figure 1:**
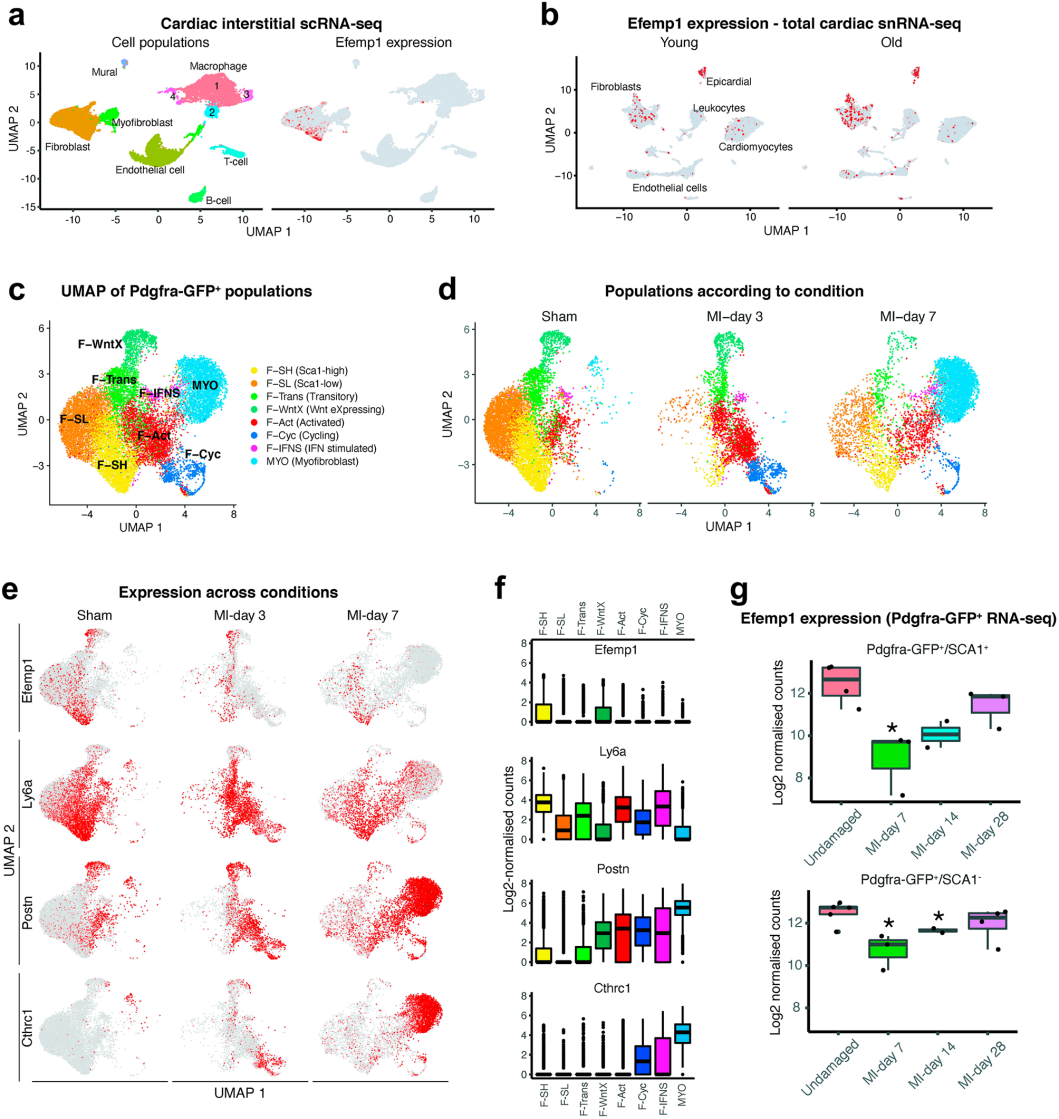
Expression of *Efemp1* in cardiac fibroblasts before and after myocardial infarction. Analysis of previously published single cell/nuclei and bulk RNA-seq datasets. (**a**) UMAP plots showing detected lineages in scRNA-seq of cardiac interstitial cells from sham and MI hearts^37^ with *Efemp1* expression indicated. (**b**) UMAP plot showing *Efemp1* expression in cardiac snRNA-seq^38^ of young vs old mice. (**c**) UMAP plot showing aggregate of *Pdgfra-*GFP^+^ cells from sham and MI hearts. (**d**) UMAP plot of GFP^+^ cells separated according to experimental condition (sham, MI-day 3, MI-day 7). (**e**,**f**) Expression of select genes in GFP^+^ cells as visualised I on UMAP coordinates according to condition or (**f**) according to box plots. (**g**) Expression of *Efemp1* in a bulk RNA-Seq MI time-course of *Pdgfra*-GFP^+^/Sca1^+^ or *Pdgfra*-GFP^+^/Sca1^-^ cells^39^. *indicates whether *Efemp1* is differentially expressed (DESeq2^19^; P_adj_ < 0.05) at an MI time-point relative to undamaged control for the indicated Sca1 fraction.

To identify the specific cellular origin(s) of fibulin-3 (*Efemp1*) in the heart, we analysed *Efemp1* expression in several single cell, single nuclei, and bulk RNA-seq datasets. In single cell RNA-seq data of interstitial cells from sham and MI hearts^12^, expression of *Efemp1* was observed specifically in fibroblasts (Fig. 1a). In total cardiac single nuclei RNA-seq^13^, *Efemp1* was observed in fibroblasts as well as epicardial cells of young and aged mice, with a distinct upregulation in fibroblasts of aged mice (Fig. 1b); expression in cardiomyocytes was sparse.

Given the predominant fibroblast expression of *Efemp1*, we further analysed single cell RNA-seq data of enriched (*Pdgfra*-GFP^+^) fibroblasts from sham hearts and hearts at 3-day and 7-day post-MI, in which numerous novel fibroblast sub-populations were previously characterised^12^. *Efemp1* was most highly expressed in two fibroblasts populations—a quiescent population marked by high expression of *Ly6a*/SCA1 (F-SH), which has progenitor cell properties and is enriched for cell adhesion genes, and an activated population expressing a strong secreted anti-WNT pathway signature (F-WntX). After MI, *Efemp1* expression was retained in F-SH and F-WntX, but was rarely detected in other activated fibroblasts, such as F-Act which are marked by *Postn* (Periostin) expression, or myofibroblasts (MYO), as marked by *Cthrc1* expression (Fig. 1c-f). Of note, overall *Efemp1* expression levels (total interstitial cells combined) were highest in sham hearts and was significantly reduced at day-3 (1.23-fold, FDR *p* = 1.79E-16) and day-7 (1.50-fold, FDR *p* = 1.82E-130), relative to sham. This is likely predominantly due to the dramatic drop in F-SH and F-WntX cell numbers present after MI (Supplementary Table 1), as *Efemp1* expression level remained unchanged in F-SH & F-WntX cells at day-3, and only a minor decrease was seen in *Efemp1* expression in F-WntX cells at day-7 (1.30-fold, FDR *p* = 0.0008), relative to sham (Supplementary Table 2). Subsequent gene co-expression analysis of this dataset (Supplementary Fig. 1) showed that the genes most highly correlated with *Efemp1* expression were markers of quiescent fibroblasts, particularly those associated with F-SH, while *Efemp1* was most negatively correlated with fibrotic genes and markers of MYO, including *Cthrc1*, *Col5a1, Col1a1* & *Postn* (Fig. 1e-f and Supplementary Fig. 1). Indeed, gene ontology biological process (GOBP) enrichment analysis revealed genes negatively correlated with *Efemp1* are over-represented for extracellular matrix organisation processes (Supplementary Table 3).

Considering a longer time course post-MI, bulk RNA-seq data of *Pdgfra*-GFP^+^/SCA1^+^ and *Pdgfra*-GFP^+^/SCA1^-^ fibroblast sub-fractions^14^ (enriched for F-SH and F-WntX cells, respectively^12^) again revealed *Efemp1* expression was highest prior injury, with *Efemp1* expression significantly reduced at day-7, but interestingly, *Efemp1* expression significantly increased again to near-uninjured levels by day 28 post-MI, in both fractions (Fig. 1g). We then compared these results to a bulk RNA-seq dataset of total cardiac cells over yet a longer a time-course out to 8 weeks post-MI^15^. Similar to data of enriched fibroblasts, *Efemp1* was downregulated early at day 1 and week 1 post-MI but was strikingly upregulated to its highest levels late at week 8 post-MI, relative to its sham control (Supplementary Fig. 1).

Collectively, this data demonstrates that *Efemp1* is expressed predominantly by fibroblast subsets enriched for adhesion and anti-WNT signatures, and that *Efemp1* expression is downregulated at early and moderate time-points post-MI, is upregulated to sham/uninjured expression levels at day 28, and is upregulated further to its highest expression levels at ~ week 8 post-MI.

### Fibulin-3 is elevated in left ventricular tissue and plasma of heart failure patients

**Figure 2:**
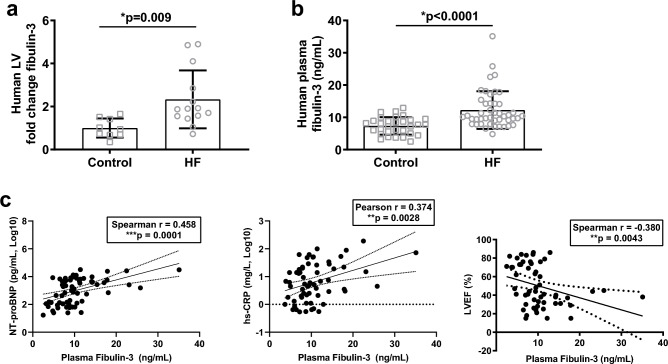
Fibulin-3 is elevated in left ventricular tissue and plasma of heart failure patients. (**a**,**b**) Fibulin-3 was significantly elevated vs controls in the ventricular tissue (LC–MS/MS analysis) and plasma of heart failure (HF) patients. (**c**) Circulating fibulin-3 in HF patients was significantly positively correlated with established biomarkers N-terminal pro b-type natriuretic peptide (NT-proBNP), high sensitivity C-reactive protein (hs-CRP), and significantly negatively correlated with LV ejection fraction (LVEF). Ventricular tissue, HF n = 15, control n = 10; Plasma n ≥ 55 including non-failing and HF patient plasma.

Next, to assess the relevance of fibulin-3 as a target for human fibrotic heart disease, we analysed fibulin-3 expression levels in left ventricle tissue and plasma from failing, and non-failing patients. Left ventricular tissue samples from end-stage ischaemic and dilated failing hearts (where fibrosis is well established), and non-failing donor hearts were subjected to LC–MS/MS analysis (failing: n = 12 ischaemic, n = 3 non-ischaemic cardiomyopathy, male 100%, age 63 ± 4, LVEF 30 ± 11%; non-failing: n = 10, male 100%, age 63 ± 3, LVEF 63 ± 4%). Fibulin-3 protein expression was two-fold higher in left ventricular tissue of heart failure patients, relative to non-failing (*p* = 0.009; Fig. 2a); in line with *Efemp1* RNA-seq analysis. Plasma was then collected from acute heart failure patients and healthy controls (failing: n = 43, male 65%, age 75 (60–81), LVEF 34.9 ± 11.8%; non-failing: n = 31, male 61%, age 67 (62–71), LVEF 74.6 ± 9.3%). Circulating fibulin-3 concentration was significantly elevated in the plasma of acute heart failure patients compared to age-matched controls (12.3 ± 5.8 vs 7.4 ± 2.7 ng/mL; *p* < 0.0001, Fig. 2b), no differences in sex were seen. As expected, levels of NT-proBNP and hs-CRP were significantly higher in the heart failure patients compared to controls (NT-proBNP 7150 ± 7745 vs 145 ± 165 pg/mL, *p* < 0.0001; hs-CRP 29 ± 37 vs 2.6 ± 3.3 mg/L, *p* < 0.0001), and circulating fibulin-3 concentration in heart failure patients was significantly positively correlated with NT-proBNP and hs-CRP, and was significantly negatively correlated with LVEF (Fig. 2c). Together, this data supports *Efemp1* RNA-seq analysis, and demonstrates that fibulin-3 is upregulated in the plasma of acute heart failure, is correlated with established measures of severity of disease, and is upregulated in the left ventricle of end-stage human heart failure patients.

### Fibulin-3 deletion increases cardiac rupture rate and ventricular remodelling post-MI

**Figure 3:**
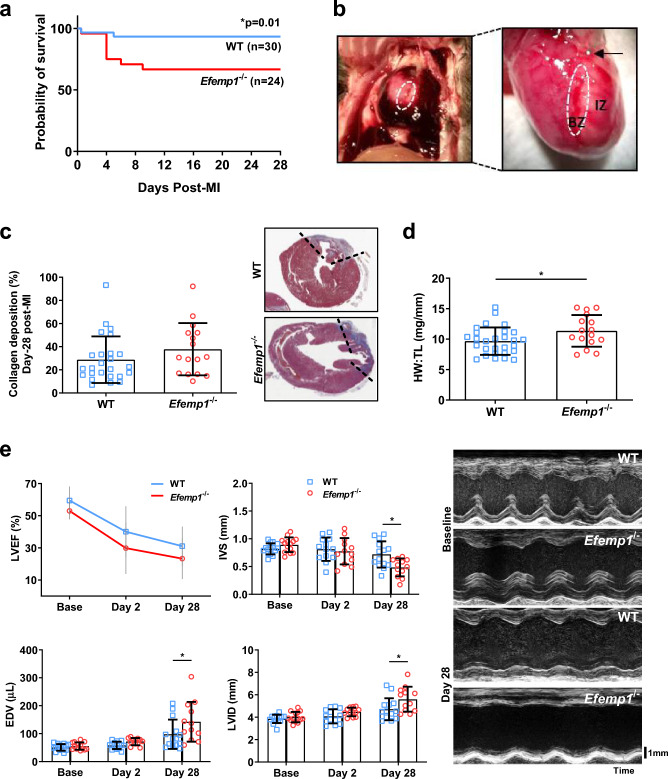
Fibulin-3 deletion results in cardiac rupture and ventricular remodelling post-infarct. (**a**) *Efemp1*^−/−^ mice had significantly lower survival rates post-MI compared to WT mice, post-mortem revealed cardiac rupture to be the predominant cause of death (n = 24 *Efemp1*^−/−^, n = 30 WT, Kaplan–Meier analysis). (**b**) Representative images of cardiac rupture post-MI along the border zone (white circle), sutured LAD is depicted by black arrow. (**c**,**d**) In the mice that survived to 28 days, collagen deposition was not different, and heart weight to tibia length ratio (HW:TL) was significantly larger in the *Efemp1*^−/−^ mice (n = 16 *Efemp1*^−/−^, n = 28 WT, *p* = 0.03). (**e**) Ejection fraction (LVEF) was not different between the groups, however ventricular remodelling was evident at 28 days post-MI—intraventricular septum thickness (IVS, *p* = 0.02), end diastolic volume (EDV, *p* = 0.001), and left ventricular internal diameter (LVID, *p* = 0.003), were significantly different between groups (n = 14 *Efemp1*^−/−^, n = 12 WT). Mean ± SD.

We then utilised a homozygous fibulin-3 genetic knockout mouse model (*Efemp1*^−/−^) which we subjected to experimental MI surgery, to investigate the functional role of fibulin-3 in cardiac fibrosis and scar formation. Survival post-MI was significantly lower in the *Efemp1*^−/−^ mice compared to WT, with only 68% of *Efemp1*^−/−^ mice surviving to 28 days, compared to 94% of WT mice (*p* = 0.01; Fig. 3a). Post-mortem analysis revealed cardiac rupture between days 3–6 to be the predominant cause of death, and the site of rupture was most frequently along the border zone (representative image, Fig. 3b). Cardiac rupture was significantly more frequent in *Efemp1*^−/−^ than in WT mice, indicating a weaker, inadequately formed scar. Male mice had a significantly higher rate of rupture than females in both *Efemp1*^−/−^ and WT mice (males vs female survival at 28 days: *Efemp1*^−/−^ 64 vs 82%; WT 93 vs 100%; *p* = 0.04, Supplementary Fig. 2). In mice that survived to 28 days, there was no significant difference in collagen deposition in the infarcted area between groups (Fig. 3c), and no significant difference in cardiomyocyte diameter between groups. However, *Efemp1*^−/−^ mice had significantly greater ventricular remodelling than WT mice as evidenced by significantly larger hearts (increased heart weight-tibia length ratio 11 ± 2.6 vs 9.7 ± 2.3 mg/mm, *p* = 0.03; Fig. 3d), significant left ventricular dilation (end diastolic volume 143 ± 71 vs 98 ± 52 µL, *p* = 0.001; LV internal diameter 5.6 ± 1.1 vs 4.7 ± 1.0 mm, *p* = 0.003), and reduced ventricular wall thickness at diastole (interventricular septum 0.48 ± 0.16 vs 0.72 ± 0.24 mm, *p* = 0.02; LV posterior wall 0.56 ± 0.29 vs 0.76 ± 0.22 mm, *p* = 0.07) (Fig. 3e). Male mice had a significantly higher end diastolic volume and heart weight-tibia length in both *Efemp1*^−/−^ and WT mice (Supplementary Fig. 3). LVEF was not significantly different at 28 days post-MI between groups of surviving mice (23 ± 13 vs 31 ± 12%), nor was there a difference between genders, although there may be some “survivor effect”. All echocardiography data is presented in Supplementary Table 4, online.

To evaluate the presence of heart failure, we analysed lung weights and pulmonary echocardiography (Supplementary Fig. 4). No difference was seen in heart:lung (*p* = 0.34), nor lung:tibia (*p* = 0.33) ratios between groups. Similarly, there was no difference in lung congestion 28 days post-MI (MoLUS score 6.5 ± 5.1 WT vs 8 ± 3.4 *Efemp1*^−/−^, *p* = 0.59).

### Fibulin-3 deletion decreases collagen content early after infarction, and may alter collagen fibre alignment

**Figure 4:**
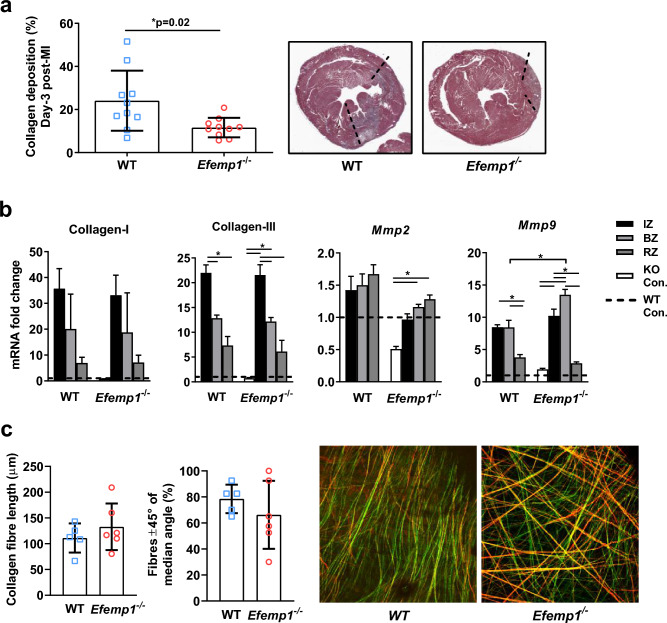
Fibulin-3 deletion resulted in less collagen content, upregulated *Mmp9* expression, and highly variable collagen organisation in the infarct zone at day-3 post-MI. (**a**) Collagen deposition was significantly reduced in the *Efemp1*^−/−^ mice at 3-days post-MI (n = 10 *Efemp1*^−/−^, n = 10 WT). (**b**) No significant differences were seen between *Efemp1*^−/−^ and WT mice in collagen-I or collagen-III mRNA expression at 3 days post-infarct, *Mmp*2 showed a trend to decreased mRNA expression, and *Mmp*9 border zone mRNA expression was significantly increased in *Efemp1*^−/−^ vs WT mice, fold change relative to WT control, dotted line; relative levels of genes normalised to a reference geomean and converted to fold change, bars represent the mean ± SD. Infarct Zone (IZ), Border Zone (IZ), and Remote Zone (RZ); all n = 5/group. (**c**) No significant difference in total group-wise collagen fibre length, nor in fibre-alignment was seen between *Efemp1*^−/−^ and WT mice at 3 days post-MI (infarct-zone tissue), however a distinct dichotomy in the *Efemp1*^−/−^ mice was seen with 2 mice displaying high fibre-alignment, and 4 with fibre disarray, n = 5–6/group. Representative images of second harmonic generation two-photon imaging (3D images displayed as maximum intensity projections) depict fibre alignment vs fibre disarray. Mean ± SD.

To explore the cause of this *Efemp1*^−/−^ cardiac rupture phenotype, we examined heart tissue at day 3 post-MI, the beginning of the period when cardiac rupture occurred, and where there was no difference in heart weight-tibia length, nor in any echocardiographic parameters. Interestingly, at 3-days post-MI there was significantly less collagen deposition in the infarct zone of *Efemp1*^−/−^ mice compared to WT (11.6 ± 4.5 vs 24.0 ± 13.2%, *p* = 0.02, Fig. 4a), demonstrating that cardiac rupture in *Efemp1*^−/−^ mice may be due to a lack of sufficient stable collagen deposition to prevent cardiac rupture post-MI. Further, there was no significant difference in *Collagen-I* and *–III* mRNA expression between WT and *Efemp1*^−/−^ mice, but mRNA expression of the gene encoding the key ECM breakdown protein, *Mmp9*, was significantly increased in the border zone (the most frequent site of rupture) of *Efemp1*^−/−^ mice compared to WT (Fig. 4b). This suggests a hypothesis that *Efemp1*^−/−^ dependent cardiac rupture may be due to an increase in rate of ECM breakdown and/or lack of ECM organisation/integrity of the infarct and surrounding border zone tissue.

To address this hypothesis, we utilised second harmonic generation two-photon imaging to assess the content, quality and alignment of collagen fibres in *Efemp1*^−/−^ and WT mice at day-3 post-MI. Fibre length in the infarct zone at day 3 post-MI was not significantly different in the *Efemp1*^−/−^ compared to WT mice (132.8 ± 45.2 vs 111.2 ± 28.3 μm, *p* = 0.38), but a high degree of fibre disarray was seen in 4 of the 6 of the *Efemp1*^−/−^ mice, compared to WT mice collagen fibres which were consistently highly aligned (Fig. 4c), supporting the hypothesis that lack of ECM organisation and an increase in ECM breakdown is the cause of cardiac rupture in *Efemp1*^−/−^ mice.

### Fibulin-3 deletion leads to differential expression of ECM organisation/integrity and inflammatory genes post-MI

**Figure 5:**
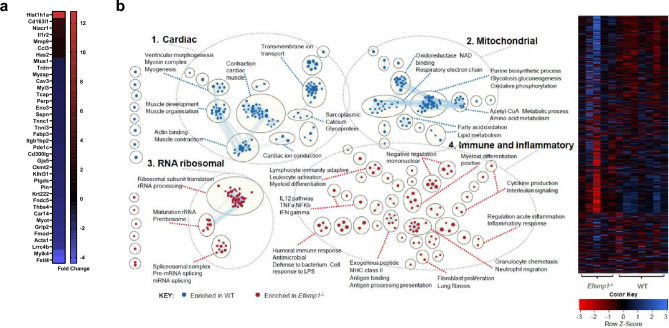
Bulk RNA-seq reveals differential expression of ECM and muscle fibre organisation genes, inflammatory genes and *Mmp9* in the infarct zone of *Efemp1*^−/−^ at day-3 post-MI. (**a**) Bulk RNA-seq was conducted on isolated infarct zone tissue from WT mice (n = 7) and *Efemp1*^−/−^ (n = 5) at day-3 post-MI. A condensed list of genes of interest are presented ranked by fold-change; edgeR analysis, all presented genes FDR ≥ 0.05. (**b**) Gene lists derived from RNA-seq underwent GSEA pathway analysis to identify pathways enriched in the WT and *Efemp1*^−/−^ mice infarct zones. Pathway enrichment results were visualized in Cytoscape using the EnrichmentMap, AutoAnnotate and ClusterMaker2 apps before being manually laid out and labels removed for visual purposes. Enriched pathways for WT are shown in blue and the pathways enriched in *Efemp1*^−/−^ mice are in red. Each node (circle) represents a distinct pathway, and edges (lines) represent overlapping genes between pathways, determined using a similarity coefficient. All the genes involved in the enriched pathways were then used to produce a heatmap, shown on the right.

Finally, to unbiasedly interrogate the effect of fibulin-3 deletion further and identify potential mechanisms of cardiac rupture in *Efemp1*^−/−^ mice post-MI, we conducted bulk RNA-seq on infarct zone tissue of *Efemp1*^−/−^ and WT mice at this early day-3 post-MI time-point. RNA-seq identified 103 genes differentially expressed in *Efemp1*^−/−^ mice relative to control. A refined list of genes of interest are presented (Fig. 5a); a list of all 103 genes including statistical data is provided in Supplementary Table 5. Of note, several key inflammatory genes were upregulated in response to *Efemp1* deletion, including Interleukin 1 Receptor Type 2 (*Il1r2*) and C–C Motif Chemokine Ligand 3 (*Ccl3)*, while several genes involved in ECM and muscle fibre assembly/organising/structural integrity were downregulated including Fibromodulin (*Fmod)*, Thrombospondin 4 (*Thbs4)* and Actin Alpha 1 *(Acta1)*. Interestingly, the key ECM breakdown protein Matrix Metalloproteinase 9 *(Mmp9)* was upregulated in *Efemp1*^−/−^ mice, but no changes in *collagen* expression were seen (in line with qPCR results in Fig. 4b). Subsequent GSEA pathway analysis revealed *Efemp1*^−/−^ mice had an overall depletion of pathways associated with heart muscle fibre assembly and contraction, myosin filament organisation, ion conduction, cardiac tissue morphogenesis, and an increase in pathways relating to inflammation, including interleukin signalling, leukocyte activation/chemotaxis, and changes to ribosomal proteins and RNA processes, relative to WT (Fig. 5B); full data including statistics are provided in Supplementary Table 6. We then used the Cytoscape search function to pull out and highlight the core genes involved in enriched and depleted GSEA pathways of interest in the *Efemp1*^−/−^ mice. Indeed, several important genes involved in scar formation following MI were identified, with *Mmp*9, *Ccl*3 and *Il1b* highly upregulated and at the top of the core enriched genes from multiple pathways (Supplementary Table 7). Together, this data strongly suggests that fibulin-3 is necessary for ECM and muscle fibre organisation, integrity, and control of ECM breakdown in wound healing post-MI, with fibulin-3 deletion leading to cardiac rupture in the infarct and border zone early post-MI.

## Discussion

Fibulin-3 is a secreted protein that is known to be involved in ECM integrity^7,8,11^. Here we provide evidence that fibulin-3 plays a novel and important role in maintaining cardiac tissue integrity after myocardial infarction. First, we demonstrated that fibulin-3 is expressed predominantly by cardiac fibroblasts enriched for adhesion (F-SH) and anti-WNT signatures (F-WntX). Second, we demonstrated that fibulin-3 is elevated in the plasma of acute heart failure patients, is correlated with established measures of severity of disease, and is upregulated in the left ventricle of end-stage human heart failure patients in which cardiac fibrosis is well established. Finally, we demonstrated that fibulin-3 deletion alters scar tissue integrity, specifically resulting in less collagen deposition and increased *Mmp9* expression early at day-3 post-MI, significantly increasing rates of death due to cardiac rupture in the border zone at days 3–6, and increasing ventricular remodelling, with no difference in collagen deposition, at day-28. Subsequent unbiased RNA-seq specifically implicated alterations in genes associated with ECM and muscle fibre organisation/integrity (*Fmod, Thbs4, Acta1*), rate of ECM breakdown (*Mmp9*) and inflammation (*Il1r2, Ccl3*) in this cardiac rupture phenotype post-MI.

*Efemp1* specific analysis of various RNAseq datasets revealed that the cellular origin of fibulin-3 expression is predominantly in cardiac fibroblasts, particularly quiescent fibroblasts enriched for adhesion and anti-Wnt signatures, rather than directly pro-fibrotic myofibroblasts (or F-Act cells). Our RNA-seq analysis clearly demonstrated that although fibulin-3 expression in fibroblasts was reduced in the interstitial cell population early following infarction (day-1 to -7), fibulin-3 was in fact upregulated to its highest levels at much later time-points, from between 28 days to 8 weeks post-MI when fibrosis is well established. This is in line with our findings in human heart failure patient left ventricle tissue where we found that fibulin-3 is significantly elevated in the left ventricle of end-stage human heart failure patients in which cardiac fibrosis is well established. Suggesting a role for fibulin-3 not in carrying out direct fibrotic tissue deposition, but rather in ECM remodelling/organisation late, and potentially interstitial fibrosis and hypertrophy/heart failure. Similarly, *Efemp1* was significantly upregulated in age-related fibrosis^13^. Late upregulation of fibulin-3 is consistent with another study in a porcine model of cardiac ischaemia–reperfusion which demonstrated significant fibulin-3 upregulation at a late time-point (60 days), with no significant change at the early time-point (15 days)^24^. Understanding these late changes will require additional single-cell expression profiling and subsequent targeted mechanistic experiments. However, these data support a role for fibulin-3 in collagen/ECM organisation which is key during these later time-points of disease (particularly MI)^25^, rather than a pro-fibrotic role in directly driving collagen deposition early. Of note, we described that the reduction in interstitial fibulin-3 expression early post-MI is likely due to the dramatic drop in the total number of *Efemp1* positive fibroblast cell populations present in the heart at these time-points, and only minorly due to the downregulation of *Efemp1* expression in F-WntX cells which occurred at day-7 (no change in *Efemp1* expression was seen at day-3). As such, conversion of fibroblasts to myofibroblasts (and other activated fibroblast subsets) at this time is likely a factor (*Efemp1* was not expressed in activated fibroblasts [F-Act] or in myofibroblasts). The later increase in *Efemp1* positive cells and overall expression level, likely corresponds to restoration of ‘unactivated’/’quiescent’ fibroblast proportions in the heart tissue at this time (e.g., F-SH & F-WntX), although we cannot presently exclude that *Efemp1* is activated in other cell types late post-MI.

We also showed that fibulin-3 was significantly upregulated in the plasma of acute heart failure patients and that fibulin-3 protein expression in these patients was significantly positively correlated with the levels of NT-proBNP (a marker of ventricular filling pressures and heart failure severity), hs-CRP (a marker of inflammation), and importantly, was negatively correlated with LVEF. Despite the routine use of NT-proBNP and hs-CRP as biomarkers for heart failure and inflammation respectively, they are not able to provide information on the underlying pathologic mechanisms. A marker that demonstrates the underlying pathology, such as myocardial fibrosis and/or relative risk of cardiac rupture, and/or potential reduction in LVEF, could be of considerable diagnostic value. It is possible that fibulin-3 plasma levels may be a valuable early-detection biomarker, reflecting the degree of underlying cardiac fibrosis. Our data are consistent with a recent study that unbiasedly identified *Efemp1* coupled with NT-proBNP in patients with acute MI was a strong predictor of secondary outcomes MACE and HF (area-under-the-curve of 0.8)^26^. Further, *Efemp1* combined with *Fstl3* specifically outperformed left ventricular measures as a benchmark prognostic factor of HF after acute MI. This provides a strong basis for future studies to further assess fibulin-3 as a biomarker post-MI to improve current prognostic benchmarks.

Our data demonstrating that fibulin-3 deficient mice have drastically increased rates of cardiac rupture after MI is powerful indication for a role of fibulin-3 in maintaining the integrity and organisation of fibrotic tissue. Cardiac rupture at days 3–6 after LAD ligation is well documented in this model, so it is not surprising that we saw ruptures during this time period in both wildtype and *Efemp1*^−/−^ mice. What is interesting is that in the absence of fibulin-3 the rate of ruptures increased from 6% in wildtype mice to 32% in *Efemp1*^−/−^. Our scRNA-Seq results demonstrated that fibulin-3 was decreased in fibroblasts at days 3 and 7 post-MI, which may contribute to the ruptures we see in wildtype mice. It is possible that the complete knockout of fibulin-3 amplifies this phenomenon. We believe that fibulin-3 is necessary for scar integrity, and that the reduced expression contributes to scar weakening and rupture.

In fact, we showed that early (day-3) post-MI fibulin-3 deficient mice had significantly less collagen deposition in the infarct, suggesting that cardiac rupture was due to a lack of sufficient stable, correctly aligned collagen in the infarct to prevent rupture. *Efemp1*^−/−^ mice who survived past the early cardiac rupture risk point, to day-28 post-MI, showed significant ventricular remodelling with dilated and thinner left ventricles despite preservation of LVEF. Strikingly, we demonstrated a dichotomy in collagen fibre-alignment within the scar tissue of *Efemp1*^−/−^ mice at 3 days post-MI, with 4 of the 6 mice displaying marked fibre disarray compared to the consistently highly aligned fibres of the WT scar, supporting a role for fibulin-3 in ECM organisation. Recent computational and experimental studies have shown that increased scar alignment (anisotropy) is a determinant of improved cardiac mechanics, function, and survival post-MI^27–31^. It is therefore enticing to speculate that the four *Efemp1*^−/−^ mice with fibre disarray may have had weaker scars that may have gone on to rupture, had they been kept longer than 3 days post-MI. Similarly, the fact that we observed significantly less collagen deposition at day-3 post-MI (the beginning of risk window for cardiac rupture, days 3–6), but not at day-28 post-MI may be due to a “survivor effect”, and thus poses a potential source of selection bias in our data at day-28, since potentially the most severe *Efemp1*^−/−^ MIs had already died due to rupture. Further investigation, however, is required to confirm this. Regardless, these results provide evidence that fibulin-3 plays a vital role in the organisation and quality, rather than quantity, of the newly generated scar tissue after MI.

Unbiased RNA-seq of infarct zone tissue investigated the potential mechanisms underpinning this cardiac rupture phenotype and shed further light on this proposed role for fibulin-3. Following MI, deletion of fibulin-3 lead to downregulation of ECM and muscle fibre assembly/organising/structural integrity genes (*Fmod, Thbs4* & *Acta1)*, but upregulation of the key ECM breakdown gene *Mmp9*; GSEA pathway analysis was supportive of this. *Mmp*9 is a gene encoding a collagenase that has been widely reported to be involved in ventricular remodelling post-infarction^32^. Correspondingly, we demonstrated a significant increase in *Mmp9* mRNA in the border zone of infarcted fibulin-3 deficient mice via qPCR. MMP-9 inhibition has been shown to reduce cardiac rupture incidence^33^, and a study in hypertensive rats demonstrated that fibulin-3 treatment significantly reduced vascular MMP9^34^. As such, increased MMP-9 expression and activity may be a key cause of this cardiac rupture phenotype. Indeed, fibulin-3 may modulate the quality of the scar via either controlling *Mmp9* mRNA expression and/or maintaining correct organisation of the ECM and muscle fibres which indirectly inhibits *Mmp9* expression, although more studies are needed to directly investigate this. On the other hand, several inflammatory genes including *Ccl3* and *Il1r2* were upregulated in *Efemp1*^−/−^ infarct zone tissue (and were top/key components in enrichment of many identified pathways), suggesting that an overstimulation in leukocyte infiltration and inflammatory pathway activation may have also significantly contributed to the underlying mechanisms of *Efemp1*^−/−^ cardiac rupture. This is in support of a recent organoid study by Sharma et al. (2022), in which we showed that there was differential expression between *Efemp1*^−/−^ and WT cardiac spheroids after ischaemia/reperfusion injury, including an upregulation of *Mmp13* and *Ccl2* and *Ccl11*^35^. Interestingly, this study also showed that control, non-infarcted *Efemp1*^−/−^cardiac spheroids demonstrated decreased expression in fibrotic genes *Col1a1*, *Ctgf*, *Dcn* and *Fn1*, with an increase in *Col1a1* and *F2r* compared to WTs. Future studies are needed to elucidate these mechanisms and should investigate the potential contribution of inflammatory mechanisms toward the function of fibulin-3 in vivo.

It is worth noting that the LAD ligation model produces mice which develop LV injury and dysfunction, but often do not develop heart failure in the short time-frame of 28 days^36^. In our study, we saw no difference in lung weights, heart:lung ratio, nor lung:tibia ratios between groups. We also saw no difference in lung congestion. Additionally, the infarct tissue RNA-Seq showed no differential expression in the NTTB gene which encodes for BNP, a marker of heart failure, suggesting that these mice did not develop heart failure, at one month post-infarct. Further studies would be required to determine if heart failure developed at a later time-point.

This study had some unavoidable limitations. First, our *Efemp1*^−/−^mice were produced as a global fibulin-3 knockout, not specific to the heart. This may have caused potential systemic changes which were not investigated in this study. Second, the infarct, border and remote zone tissue was defined as including 100%, 50%, and 0% infarcted tissue, respectively, however it is possible that there was slight variation between tissue samples; investigators were blinded to genotype when collecting the tissue to prevent unintended bias. Third, our study demonstrated that MMP9 may play a key mechanistic role, however MMP activity was not ascertained in this study. Finally, the human LV tissue was only available in men, preventing a gender analysis.

Collectively, our study provides evidence that fibulin-3 is necessary for ECM organisation, integrity, and scar formation post-MI, with fibulin-3 deletion leading to a significant phenotype of cardiac rupture in the border zone early post-MI. Thus, fibulin-3 has an important and novel role in cardiac fibrosis and specifically quality and integrity of fibrotic tissue formation with cardiac disease.

### Supplementary Information

Supplementary Information.

## Data Availability

All data generated or analysed during this study are included in this published article (and its Supplementary Information file) or are available from the corresponding author on reasonable request.
