# Supplementary material for: Fibulin-3 is necessary to prevent cardiac rupture following myocardial infarction
Source: Sci Rep. 2023 Sep 11;13:14995. doi: 10.1038/s41598-023-41894-9 (PMC10495317; doi:10.1038/s41598-023-41894-9)
Supplement: Supplementary file 1 — Supplementary Information. [file 41598_2023_41894_MOESM1_ESM.pdf]

SUPPLEMENTARY INFORMATION

Fibulin-3 is necessary to prevent cardiac rupture following myocardial infarction

Lucy A. Murtha, PhD [1,2], Sean A. Hardy, PhD [1,2], Nishani S. Mabotuwana, B Biomed Sci [1,2], Mark J. Bigland, PhD [1,2], Taleah Bailey, B Biomed Sci [1,2], Kalyan Raguram, MBBS [1,2], Saifei Liu, MD, PhD [3], Doan T. Ngo, PhD [1,2,3], Aaron L. Sverdlov, MBBS, PhD [1,2,3,4], Tamara Tomin, PhD [5,6], Ruth Birner-Gruenberger, PhD [5,6], Robert D. Hume, PhD [7], Siiri E. Iismaa, PhD [8,9], David T. Humphreys, PhD [8,9], Ralph Patrick, PhD [8,9], James J.H. Chong, MBBS, PhD [7,10,11], Randall J. Lee, MD, PhD [12,13,14], Richard P. Harvey, PhD [8,9,15], Robert M. Graham, MBBS, MD [8,9], Peter P. Rainer, MD, PhD [16], Andrew J. Boyle*, MBBS, PhD [1,2,4]

1. Faculty of Health and Medicine, The University of Newcastle, Newcastle, NSW, Australia
2. Hunter Medical Research Institute, Newcastle, NSW, Australia
3. Department of Cardiology and Clinical Pharmacology, Basil Hetzel Institute, The University of Adelaide, The Queen Elizabeth Hospital, Adelaide, SA, Australia
4. Department of Cardiovascular Medicine, John Hunter Hospital, Newcastle, NSW, Australia
5. Institute of Chemical Technologies and Analytics, Faculty of Technical Chemistry, Technische Universität Wien, Vienna, Austria
6. Diagnostic and Research Institute of Pathology, Medical University of Graz, Graz, Austria
7. Centre for Heart Research, Westmead Institute for Medical Research, The University of Sydney, Sydney, NSW, Australia
8. Victor Chang Cardiac Research Institute, Sydney, NSW, Australia
9. St Vincent's Clinical School, UNSW, Sydney, Kensington, NSW, Australia
10. Department of Cardiology, Westmead Hospital, Sydney, NSW, Australia
11. Faculty of Medicine and Health, The University of Sydney, Sydney, NSW, Australia
12. Department of Medicine, Division of Cardiology, University of California San Francisco, San Francisco, CA, USA
13. Edyth and Eli Broad Center for Regenerative Medicine and Stem Cell Research, University of California San Francisco, San Francisco, CA, USA
14. Cardiovascular Research Institute, University of California San Francisco, San Francisco, CA, USA
15. School of Biotechnology and Molecular Bioscience, UNSW, Sydney, Kensington, NSW, Australia
16. Division of Cardiology, Medical University of Graz, Graz, Austria and BioTechMed Graz, Graz, Austria

Short Title: Fibulin-3, crucial to prevent cardiac rupture post-infarct

Address for correspondence:
Andrew J. Boyle, MBBS, PhD
Hunter Medical Research Institute, Kookaburra Circuit, Newcastle, NSW 2305, Australia
Email: Andrew.boyle@newcastle.edu.au Phone: +61249214205

Supplementary Methods

1. *Analysis of online single cell/nuclei and bulk RNA-Seq datasets*

Online single-cell (sc)RNA-Seq and single-nuclei (sn)RNA-Seq datasets are available from ArrayExpress (www.ebi.ac.uk/arrayexpress) under accession codes E-MTAB-7376 (scRNA-Seq of interstitial and *Pdgfra*-GFP$^+$ cardiac cells from sham and MI hearts [1]) and E-MTAB-7869 (snRNA-Seq of young and aged healthy mouse hearts [2]). Bulk RNA-Seq datasets are available from the gene expression omnibus (www.ncbi.nlm.nih.gov/geo) under identifiers GSE141929 (*Pdgfra*-GFP$^+$ cardiac cells from uninjured and MI hearts [3]) and GSE114695 (total cardiac cells from the left ventricles of sham and MI hearts [4]).

scRNA-Seq and snRNA-Seq datasets were analysed using the *Seurat* version 3.1.4 R package [5]. The scRNA-Seq datasets were processed to log-normalized counts, scaled and principal component (PC) analysis performed on the top 2000 variable genes. For snRNA-Seq, the replicates were batch aligned, similar to previously [2], using the *FindIntegrationAnchors* and *IntegrateData* functions in Seurat and scaled prior to PC analysis. For all datasets, UMAP dimensionality reduction was run on the top 25 PCs. Cell labels were assigned to *Pdgfra*-GFP$^+$ cells using previous characterisations [1]. For the corresponding interstitial cell dataset, the *FindNeighbors* function in *Seurat* was run on the top 25 PCs, followed by *FindClusters* with the *res* parameter set to 0.2. Gene co-expression analysis with *Efemp1* was performed by calculating Pearson correlation coefficients between *Efemp1* and all other genes on log-normalized counts in the *Pdgfra*-GFP$^+$ dataset and selecting the top 20 most positively and negatively correlated genes.

For the GSE141929 bulk RNA-seq, previous gene counts were available and downloaded for analysis. For GSE114695, raw Fastq files were downloaded, mapped and processed to gene counts using STAR

aligner [6] following removal of Illumina adaptors and trimming of low-quality bases using Trimmomatic [7]. Analysis of bulk RNA-Seq data was performed using DESeq2 [8]. Differentially expressed (DE) genes were evaluated between MI time-points and undamaged hearts for the Pdgfra-GFP^{+} RNA-Seq, and between MI and sham hearts for matched time-points in the total cardiac cell RNA-Seq. *Efemp1* was considered DE if it obtained an adjusted p-value below 0.05.

2. *LC-MS/MS analysis*

Failing and non-failing heart tissue specimens were lysed in 200 µl of 100 mM Tris pH = 8.5, containing 1 % sodium dodecylsulfate and 10 mM of NEM, and 100 µg of protein was acetone-precipitated overnight. Protein pellets were re-dissolved in 87.5 µl of 50 % trifluoroethanol in 50 mM ammonium-bicarbonate, reduced with tris(2-carboxyethyl)phosphine (5 mM final concentration), then re-alkylated and digested with trypsin overnight at 37°C. The tryptic digest (40 µg) was then fractionated using Pierce High pH Reversed-Phase Peptide Fractionation Kit (Thermo Fisher, USA) according to manufacturer's instructions and subjected to LC-MS/MS analysis. Chromatography was carried out on an Ultimate 3000 RCS Nano Dionex system equipped with an Ionoptiks Aurora Series UHPLC C18 column (250 mm x 75 µm, 1.6 µm) (Bruker Daltonics, Germany). Mass spectrometry was performed on a Maxis II qTOF set to fragment the top 20 most abundant peptides.

3. RNA isolation and analysis of day 3 post-MI tissue

Quantitative polymerase Reaction (qPCR) was performed to determine the expression levels of a subset of genes belonging to four genes associate with scar formation (Collagen-I alpha-1, Collagen-III alpha-1, Mmp2 & Mmp9). RNA isolation was conducted using TRIzol® Plus RNA purification reagents (Life Technologies) as per the manufacturer protocol. RNA samples were subject to DNase I treatment using a DNase I, Amplification Grade Kit (ThermoFisher scientific) as per the manufacturer protocol.

Total RNA was quantified using the NanoDrop 1000 then DNAse 1 treated (Invitrogen). One µg of RNA was used for the synthesis of cDNA using Oligo (dT)18 primers (Bioline), dNTP (Bioline) and a SuperScript® III (Invitrogen) First Strand Synthesis System for RT-PCR.

Assays were validated by generating standard curves to evaluate the efficiency of each primer set. The specificity of PCR products was analysed via melt curve and gel electrophoresis. All target and reference genes from cDNA transcripts were measured using quantitative real-time polymerase chain reaction (qPCR), using the 7500 SDS software v2.0.6 (Applied Biosystems, Australia), and were performed on the ABI 7500. The geomean of housekeeping genes Hprt and TPt1 mRNA abundance was used as the reference, and final values calculated using the ΔΔCt method. The following primers were used:

**target**	**NCBI Gene Sequence Reference**	**FW primer**	**REV primer**
Tpt1	NM_009429.3	ATGACGAGCTGTTCTCCGAC	AACACCGGTGACTACTGTGC
Hprt	NM_013556.2	ACAGGCCAGACTTTGTTGGA	ACTTGCGCTCATCTTAGGCT
Col3a1	NM_009930.2	GCGGCTGAGTTTTATGACGG	GGTGGCTGCATCCCAATTCA
Col1a1	NM_007742.4	CTGACGCATGGCCAAGAAGA	TACCTCGGGTTTCCACGTCT
Mmp9	NM_013599.5	CCAGAGGTAACCCACGTCAG	TGGAAACTCACACGCCAGAA
Mmp2	NM_008610.3	GATAACCTGGATGCCGTCGT	TGGTGTGCAGCGATGAAGAT

4. Second harmonic generation two-photon imaging

Imaging was carried out on a Leica TCS SP8 MP microscope using 800 nm excitation laser wavelength. Collagen fibre orientation and length was quantified on Fiji Image J [9]. Brightness and exposure threshold settings were applied to all imaged samples, and 40 collagen fibres were traced per image. The proportion of fibres within ±30° of the median angle for each animal was assessed to determine the degree of fibre-alignment. Analysis was performed by two blinded investigators with strong inter-assessor reliability (length: $r=0.917$, $p<0.0001$; angle: $r=0.963$, $p<0.0001$).

5. *Gene set enrichment analysis*

GSEA pre-ranked run parameters used the base settings with geneset permutation and 1000 permutations. Prior to analysis, a ranked list was calculated with each gene assigned a score based on the FDR and the direction of the log fold-change ("+" or "−"). Results of the GSEA were then loaded into Cytoscape version 3.7.2 for visualisation and further analysis using the EnrichmentMap plugin. The settings used in Cytoscape were Jaccard Overlap Combined: 0.375 - Test used: Jaccard Overlap Combined Index (k constant = 0.5). A permutation-based P value that was corrected for multiple testing to produce a permutation-based false discovery rate (FDR) Q-value with a cutoff of 0.01 for initial analysis and increased to 0.001 for image visualisation. Nodes were coloured to reflect the different groups in question, and networks of related ontologies were circled and assigned a group label using the AutoAnnotate application. The network map layout algorithm used was prefuse force-directed weighted using the gene set similarity coefficient followed by manual adjustments, separating networks and nodes by direction of fold-change for clarity purposes.

Supplementary Figures and Tables

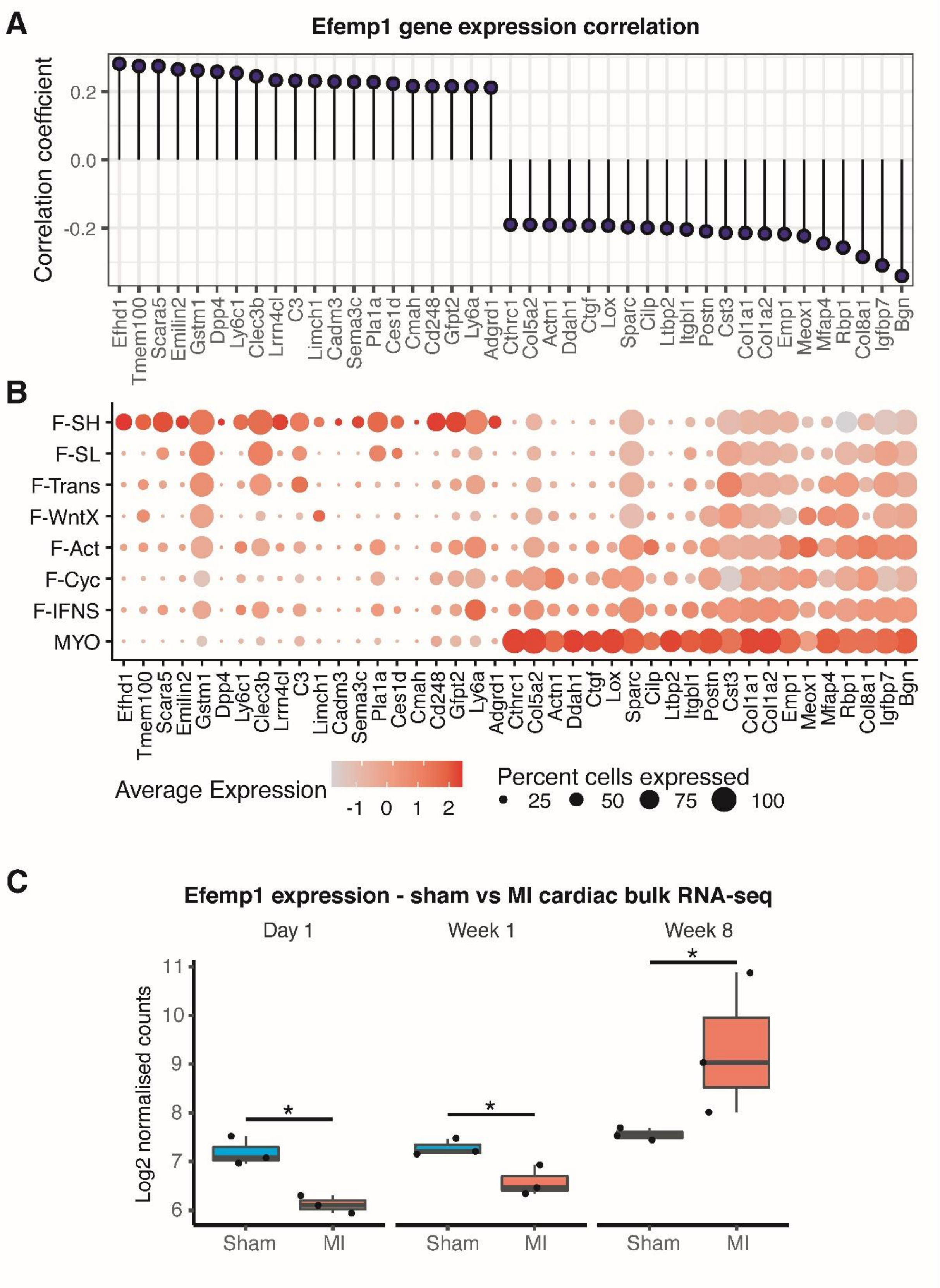
A
Efemp1 gene expression correlation
Correlation coefficient
0.2
0.0
-0.2
Efhd1 Tmem100 Scara5 Emilin2 Gstm1 Dpp4 Ly6c1 Clec3b Lrrn4cl C3 Limch1 Cadm3 Sema3c Pla1a Ces1d Cmah Cd248 Gfpt2 Ly6a Adgrd1 Cthrc1 Col5a2 Actn1 Ddah1 Ctgf Lox Sparc Cilp Ltbp2 Itgbl1 Postn Cst3 Col1a1 Col1a2 Emp1 Meox1 Mfap4 Rbp1 Col8a1 Igfbp7 Bgn
B
F-SH
F-SL
F-Trans
F-WntX
F-Act
F-Cyc
F-IFNS
MYO
Average Expression
-1 0 1 2
Percent cells expressed
25 50 75 100
C
Efemp1 expression - sham vs MI cardiac bulk RNA-seq
Day 1
Week 1
Week 8
Log2 normalised counts
Sham
MI
*

Supplementary Figure 1. (A) Top positively and negatively correlated genes with *Efemp1* in *Pdgfra*-GFP^{+} cells [10]. (B) Expression of top positively and negatively correlated genes according to a dot plot where colour indicates level of expression and size of the dot indicates the percentage of cells expressing the gene. (C) Expression of *Efemp1* in a bulk RNA-seq MI time course comparing MI or sham hearts at day 1, week 1 and week 8 post-injury [4]. *indicates whether *Efemp1* is differentially expressed (DESeq2; $P_{adj} < 0.05$) at an MI time-point relative to its sham control.

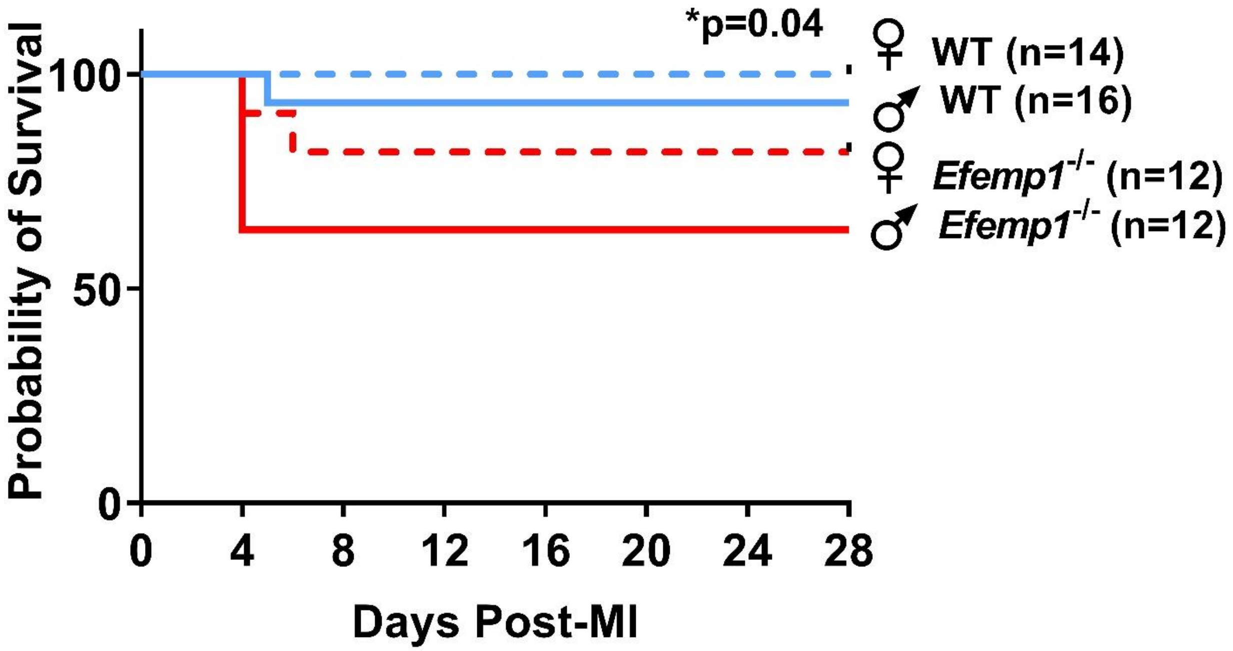

Supplementary Figure 2. Fibulin-3 deficient male mice had significantly higher rates of cardiac rupture than females. Higher rates of rupture in male mice has been reported in the literature [11-14], with one paper demonstrating 2-3 fold increased rupture in 3 strains of male mice [11]. Intramural haemorrhage severity, hematoma formation and inflammatory cell accumulation were suggested potential mechanisms. Another study indicated estrogenic as a potential mechanism, demonstrating that estrogenic-treated male mice had reduced cardiac rupture prevalence, which was associated with reduced MMP-9 activity, and AKT pathway apoptosis inhibition [12]. A further study suggested increases in the severity of inflammation, MMP-9 activation and damage to collagen matrix accounted for the male bias in cardiac rupture [13]. The mechanisms governing the gender differences seen in rupture prevalence was outside the scope of this study, however investigation into the inflammatory, MMP, and collagen matrix differences are warranted.

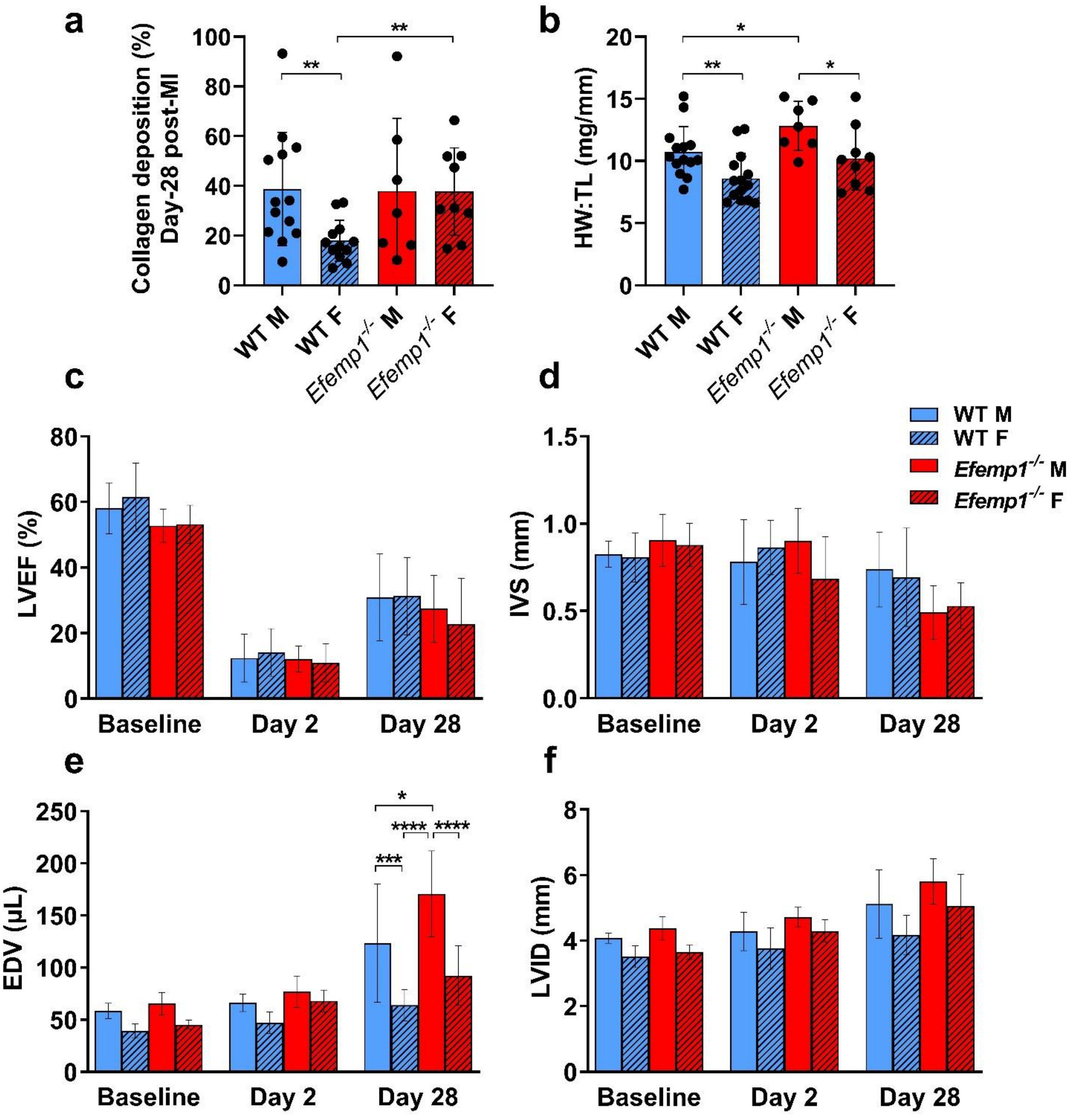

Supplementary Figure 3. Gender differences in WT and *Efempl*$^{-/-}$ mice post-MI. Histology and echocardiography data show there was no difference between genders for collagen deposition (a), LVEF (c), IVS (d) or LVID (e), however males had significantly higher EDV and HW:TL (b, e). Heart weight to tibia length ratio (HW:TL) and collagen deposition: n=16 *Efemp1*$^{-/-}$, n=28 WT. Ejection fraction (LVEF), intraventricular septum thickness (IVS), end diastolic volume (EDV), left ventricular internal diameter (LVID): n=14 Efemp1-/-, n=12 WT. Mean ± SD.

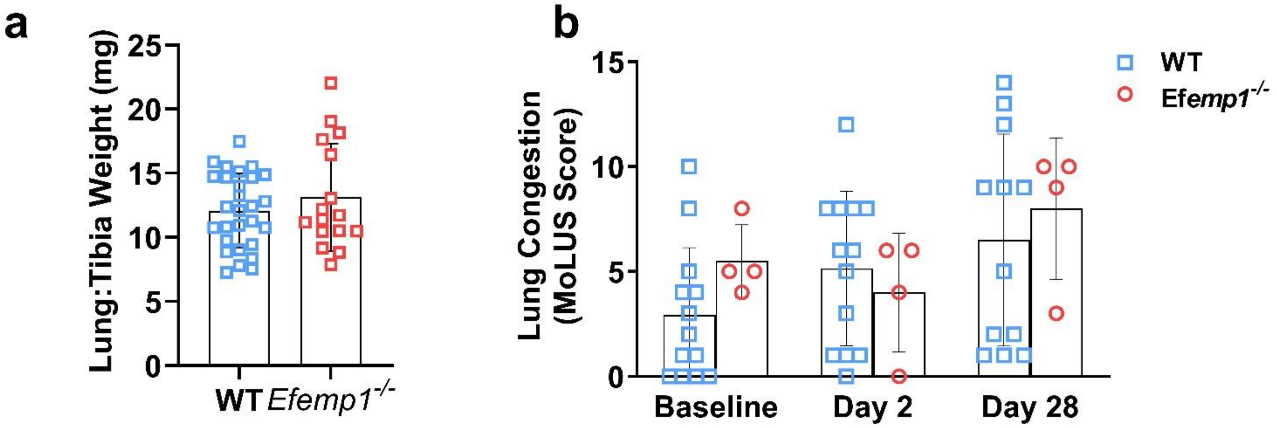

Supplementary Figure 4. Pulmonary Analysis in WT and *Efempl*$^{-/-}$ mice post-MI. To assess the development of lung congestion as a marker of heart failure, lungs were weighed (a), and assessed with echocardiography (b). MoLUS – Mouse Lung UltaSound [15]. Mean ± SD.

Supplementary Table 1. Cell number and proportion metadata of interstitial cell populations. Data is separated by time-point/condition post-MI, from the mouse scRNAseq dataset [16].

Rank	Cluster	Condition	Cell #	% total cells at time
1	MYO	Sham	22	0.384413769
2	MAC8	Sham	13	0.227153591
3	F-SH	Sham	657	11.47999301
4	F-Act	Sham	553	9.662764284
5	F-WntX	Sham	96	1.677441901
6	M1MΦ	Sham	3	0.052420059
7	MAC-IFNIC	Sham	3	0.052420059
8	F-SL	Sham	1348	23.55408003
9	M2MΦ	Sham	26	0.454307182
10	Mural	Sham	184	3.215096977
11	M1Mo	Sham	40	0.698934125
12	EC3	Sham	229	4.001397868
13	DC	Sham	24	0.419360475
14	MAC-TR	Sham	159	2.778263149
15	EC2	Sham	241	4.211078106
16	Cyc	Sham	37	0.646514066
17	Glial	Sham	18	0.314520356
18	EC1	Sham	1215	21.23012406
19	MAC6	Sham	33	0.576620654
20	TC1-Cd8	Sham	152	2.655949677
21	MAC7	Sham	44	0.768827538
22	NKC	Sham	48	0.838720951
23	TC2-Cd4	Sham	132	2.306482614
24	BC	Sham	446	7.793115499
			**Total # cells present**	**5723**
**Rank**	**Cluster**	**Condition**	**Cell #**	**% total cells at time**
1	MAC8	MI-day 3	106	2.735483871
2	MYO	MI-day 3	4	0.103225806
3	F-SH	MI-day 3	25	0.64516129
4	F-SL	MI-day 3	40	1.032258065
5	M1MΦ	MI-day 3	1964	50.68387097
6	M2MΦ	MI-day 3	49	1.264516129
7	F-WntX	MI-day 3	8	0.206451613
8	MAC-IFNIC	MI-day 3	125	3.225806452
9	F-Act	MI-day 3	185	4.774193548
10	Cyc	MI-day 3	71	1.832258065
11	M1Mo	MI-day 3	493	12.72258065

12	MAC6	MI-day 3	110	2.838709677
13	Mural	MI-day 3	10	0.258064516
14	EC2	MI-day 3	37	0.95483871
15	MAC-TR	MI-day 3	23	0.593548387
16	EC3	MI-day 3	75	1.935483871
17	EC1	MI-day 3	162	4.180645161
18	MAC7	MI-day 3	43	1.109677419
19	TC2-Cd4	MI-day 3	16	0.412903226
20	TC1-Cd8	MI-day 3	28	0.722580645
21	DC	MI-day 3	271	6.993548387
22	BC	MI-day 3	27	0.696774194
23	NKC	MI-day 3	2	0.051612903
24	Glial	MI-day 3	1	0.025806452
			**Total # cells present**	**3875**
**Rank**	**Cluster**	**Condition**	**Cell #**	**% total cells at time**
1	MAC8	MI-day 7	18	0.482185909
2	F-WntX	MI-day 7	15	0.401821591
3	MYO	MI-day 7	429	11.49209751
4	F-SH	MI-day 7	172	4.607554246
5	F-Act	MI-day 7	368	9.858023038
6	F-SL	MI-day 7	265	7.098848111
7	M1MΦ	MI-day 7	134	3.589606215
8	Mural	MI-day 7	39	1.044736137
9	MAC-IFNIC	MI-day 7	27	0.723278864
10	M2MΦ	MI-day 7	600	16.07286365
11	EC3	MI-day 7	102	2.73238682
12	Cyc	MI-day 7	108	2.893115457
13	EC2	MI-day 7	95	2.544870078
14	M1Mo	MI-day 7	79	2.11626038
15	MAC-TR	MI-day 7	55	1.473345834
16	EC1	MI-day 7	503	13.47441736
17	MAC6	MI-day 7	26	0.696490758
18	DC	MI-day 7	96	2.571658184
19	NKC	MI-day 7	26	0.696490758
20	TC2-Cd4	MI-day 7	95	2.544870078
21	TC1-Cd8	MI-day 7	144	3.857487276
22	BC	MI-day 7	278	7.44709349
23	MAC7	MI-day 7	54	1.446557728
24	Glial	MI-day 7	5	0.13394053
			**Total # cells present**	**3733**

Supplementary Table 2. *Efemp1* expression level metadata of interstitial cell populations. Data is separated by time-point/condition post-MI, from the mouse scRNAseq dataset [16].

Cell type	Comparison	p-value	avg_log2FC	FC (fold hange)	pct.1	pct.2	p-value adj.
All-cells	MI-day 3 vs sham	1.16E-20	-0.292960245	-1.225151571	0.171	0.231	1.79E-16
F-Act	MI-day 3 vs sham	5.73E-08	0.289387695	1.222121477	0.128	0.053	0.000881057
F-SL	MI-day 3 vs sham	0.057302162	0.263363636	1.200273877	0.198	0.142	1
F-SH	MI-day 3 vs sham	0.075556715	0.200029932	1.148722188	0.457	0.43	1
F-WntX	MI-day 3 vs sham	0.163198825	-0.123439616	-1.089328903	0.344	0.411	1
F-Trans	MI-day 3 vs sham	0.080210514	-0.111755944	-1.080542594	0.125	0.125	1
MYO	MI-day 3 vs sham	0.116412928	0.401389359	1.320779248	0.167	0.014	1
F-Cyc	MI-day 3 vs sham	0.031441644	-0.426584976	-1.344048293	0.107	0.19	1
All-cells	MI-day 3 vs sham	0.02143049	-0.511474719	-1.4255066	0.061	0.19	1
F-IFNS	MI-day 3 vs sham	NA	NA	#VALUE!	NA	NA	NA
**Cell type**	**Comparison**	**p-value**	**avg_log2FC**	**FC (fold hange)**	**pct.1**	**pct.2**	**p-value adj.**
All-cells	MI-day 7 vs sham	1.18E-134	-0.581584102	-1.496491518	0.077	0.231	1.82E-130
F-WntX	MI-day 7 vs sham	5.40E-08	-0.373791968	-1.295754108	0.138	0.411	0.000830452
F-IFNS	MI-day 7 vs sham	3.21E-06	-0.789749051	-1.728773725	0.038	0.26	0.049354064
F-SL	MI-day 7 vs sham	0.01049345	-0.093937297	-1.067278951	0.105	0.142	1
F-SH	MI-day 7 vs sham	0.02247654	-0.155479678	-1.113791879	0.374	0.43	1
F-Cyc	MI-day 7 vs sham	0.02143049	-0.511474719	-1.4255066	0.061	0.19	1
All-cells	MI-day 7 vs sham	0.02143049	-0.511474719	-1.4255066	0.061	0.19	1
F-Act	MI-day 7 vs sham	NA	NA	#VALUE!	NA	NA	NA
F-Trans	MI-day 7 vs sham	NA	NA	#VALUE!	NA	NA	NA
MYO	MI-day 7 vs sham	NA	NA	#VALUE!	NA	NA	NA

Supplementary Table 3. GOnet/DICE GOBP enrichment of genes negatively correlated with *Efemp1* expression presented in Supplementary Fig 1A, from the mouse scRNAseq dataset [16].

Rank	GO_term_ID	GOBP term	p-value	FDR p-value	# of genes
1	GO:0043062	extracellular structure organization	0.00E+00	1.75E-07	8
2	GO:0030198	extracellular matrix organization	0.00E+00	1.24E-07	8
3	GO:0097435	supramolecular fiber organization	2.00E-09	7.18E-06	8
4	GO:0030199	collagen fibril organization	7.75E-08	2.11E-04	4
5	GO:0085029	extracellular matrix assembly	4.98E-07	1.09E-03	3
6	GO:0001503	ossification	9.36E-07	1.70E-03	5
7	GO:0048513	animal organ development	1.67E-06	2.60E-03	12
8	GO:0007155	cell adhesion	2.56E-06	3.06E-03	7
9	GO:0001501	skeletal system development	2.68E-06	3.06E-03	6
10	GO:0022610	biological adhesion	2.80E-06	3.06E-03	7
11	GO:0009653	anatomical structure morphogenesis	5.23E-06	5.19E-03	10
12	GO:0048731	system development	7.18E-06	6.52E-03	13
13	GO:0048251	elastic fiber assembly	2.43E-05	2.04E-02	2
14	GO:0007275	multicellular organism development	3.47E-05	2.56E-02	13
15	GO:0043589	skin morphogenesis	3.72E-05	2.56E-02	2
16	GO:0061448	connective tissue development	3.76E-05	2.56E-02	4
17	GO:0009888	tissue development	4.47E-05	2.87E-02	8
18	GO:0110148	biomineralization	5.62E-05	3.13E-02	3
19	GO:0031214	biomineral tissue development	5.62E-05	3.13E-02	3
20	GO:0016043	cellular component organization	5.74E-05	3.13E-02	13
21	GO:0001568	blood vessel development	6.43E-05	3.34E-02	5
22	GO:0048856	anatomical structure development	7.99E-05	3.80E-02	13
23	GO:0071840	cellular component organization or biogenesis	8.54E-05	3.80E-02	13
24	GO:0001944	vasculature development	8.71E-05	3.80E-02	5
25	GO:0048646	anatomical structure formation involved in morphogenesis	8.72E-05	3.80E-02	6
26	GO:0072358	cardiovascular system development	9.78E-05	4.10E-02	5
27	GO:0007160	cell-matrix adhesion	1.06E-04	4.26E-02	3
28	GO:0070208	protein heterotrimerization	1.15E-04	4.48E-02	2

Supplementary Table 4. Echocardiography parameters following experimental myocardial infarction.

	**Baseline**		**Day 2**		**Day 28**	
	**WT**	***Efemp1$^{-/-}$***	**WT**	***Efemp1$^{-/-}$***	**WT**	***Efemp1$^{-/-}$***
Weight (g)	26.2 ± 4.4	27.6 ± 5	26 ± 4.1	27.4 ± 4.9	26.9 ± 4.1	28.1 ± 6
Heart Rate (BPM)	448 ± 55	470 ± 44	578 ± 43	556 ± 59	521 ± 56	533 ± 51
LVEF (%)	57.5 ± 8	55.4 ± 7.9	38 ± 15.1	33.5 ± 15.5	31 ± 12.2	23.3 ± 12.7
SV (µL)	29.7 ± 5.8	29.3 ± 7.5	21.8 ± 7.3	21.7 ± 9.4	26.9 ± 10	29.8 ± 16.3
FS (%)	19.8 ± 5	19.6 ± 4.5	13 ± 5.8	11.8 ± 4.9	8.3 ± 3.1	10.2 ± 6.1
CO (mL/min)	13.4 ± 3.5	13.7 ± 3.5	12.5 ± 3.9	12.1 ± 5.2	14 ± 5.6	15.6 ± 8.5
FAC (%)	50.8 ± 10.5	47.8 ± 10.6	36.2 ± 17.2	32.2 ± 15.3	32 ± 13.8	22.2 ± 13.7
MV E/A	1.29 ± 0.25	1.43 ± 0.31	1.39 ± 0.33	1.26 ± 0.17	1.28 ± 0.26	1.39 ± 0.29
EDV (µL)	52.3 ± 10.2	53.2 ± 12.1	60.6 ± 14.8	68.7 ± 18	97.9 ± 52.4	142.6 ± 71.4
ESV (µL)	22.6 ± 7	23.9 ± 7.2	38.8 ± 16.1	46.9 ± 19	71 ± 46	112.8 ± 68.2
LVID;d (mm)	3.9 ± 0.4	3.9 ± 0.4	4.1 ± 0.5	4.3 ± 0.5	4.7 ± 1	5.6 ± 1.1
LVID;s (mm)	2.8 ± 0.5	2.8 ± 0.5	3.3 ± 0.8	3.7 ± 0.7	4 ± 1.2	5.1 ± 1.4
IVS;d (mm)	0.8 ± 0.1	0.9 ± 0.1	0.8 ± 0.3	0.7 ± 0.2	0.7 ± 0.2	0.5 ± 0.2
IVS;s (mm)	1.2 ± 0.3	1.1 ± 0.3	1 ± 0.4	0.9 ± 0.3	0.9 ± 0.4	0.6 ± 0.3
LVPW;d (mm)	0.8 ± 0.1	0.8 ± 0.1	0.8 ± 0.2	0.7 ± 0.2	0.8 ± 0.2	0.6 ± 0.3
LVPW;s (mm)	1.1 ± 0.2	1.1 ± 0.2	1 ± 0.3	0.9 ± 0.3	1 ± 0.3	0.7 ± 0.4
LV Mass (mg)	90.1 ± 16.9	93.3 ± 24.3	96.1 ± 27.7	95.3 ± 29.6	113.2 ± 45.1	94.7 ± 30.9
MoLUS score	2.92 ± 3.22	5.50 ± 1.73	5.15± 3.69	4.00 ± 2.82	6.50 ± 5.05	8.00 ± 3.37

LV ejection fraction (LVEF) and end-diastolic volume was assessed in the parasternal 2D long-axis view. LV internal diameter and ventricular wall thickness (intraventricular septal wall; LV posterior wall) were obtained at the mid-papillary short-axis view. SV, stroke volume; FS, fractional shortening; CO, cardiac output; FAC, fractional area change; EDV, end diastolic volume; ESV, end systolic volume; LVID, left ventricular internal diameter; IVS, intraventricular septum; LVPW, left ventricular posterior wall; d, diastolic; s, systolic; LV, left ventricle; MoLUS, mouse lung ultrasound. Mean ± SD

Supplementary Table 5. Full list of differentially expressed genes from bulk RNA-seq of infarct zone tissue of *Efemp1*$^{-/-}$ and WT mice at day-3 post-MI, ranked by fold change of expression in *Efemp1*$^{-/-}$ mice relative to WT.

Gene #	Gene	Chr	P-value	FDR	Fold change
1	Hist1h1a	13	4.09E-05	0.023708	12.79891137
2	Hist1h2bb	13	8.1E-06	0.008095	11.63779796
3	Hist1h1b	13	9.99E-05	0.037073	10.58158179
4	Hist1h2b	13	9.74E-05	0.036688	9.852124096
5	Hist1h4d	13	0.000125	0.04011	6.642597966
6	Fosb	7	1.73E-05	0.013336	5.558015277
7	Cd163l1	7	4.9E-05	0.024229	3.113529611
8	Niacr1	5	4.48E-05	0.023708	3.112720416
9	Il1r2	1	6.99E-05	0.032411	2.568681649
10	Gsta3	1	4.14E-05	0.023708	2.032256637
11	Mmp9	2	0.000174	0.046484	1.66138093
12	Nupr1	7	0.000191	0.048063	1.641822769
13	Ccl3	11	0.000162	0.045792	1.607002251
14	Osgin1	8	0.000117	0.038906	1.591658202
15	Has2	15	0.000182	0.046799	1.579222938
16	Rdh10	1	0.000173	0.046484	1.44114576
17	Gpr146	5	0.000144	0.043809	-1.29699864
18	Mtus1	8	8.97E-05	0.03518	-1.32776045
19	Smarcd3	5	0.000146	0.043809	-1.37198973
20	Trdn	10	0.00015	0.043809	-1.37372557
21	Slc4a3	1	8.18E-05	0.03373	-1.40470187
22	Fam174b	7	4.56E-05	0.023708	-1.41929322
23	Myzap	9	0.000151	0.043809	-1.44083237
24	Cav3	6	3.4E-05	0.022668	-1.48632015
25	Ppif	14	0.000148	0.043809	-1.49431931
26	mt-Nd5	M	9.87E-06	0.009493	-1.50034876
27	Gmpr	13	4.69E-06	0.005538	-1.50277571
28	Lpl	8	0.000116	0.038906	-1.50944899
29	Ldhb	6	1.75E-05	0.013336	-1.51587033
30	Art3	5	4.94E-05	0.024229	-1.51723736
31	Acsl1	8	0.000115	0.038906	-1.52112494
32	Myl3	9	0.000172	0.046484	-1.54089793
33	Apobec2	17	7.34E-05	0.032867	-1.54199544
34	Tcap	11	1.41E-05	0.011613	-1.55541652
35	Perp	10	4.07E-05	0.023708	-1.56865077
36	Eno3	11	0.00012	0.039107	-1.56872324
37	Sspn	6	8.59E-05	0.034856	-1.57093327

38	Tnnc1	14	0.000104	0.037511	-1.57425575
39	Dgat2	7	0.000104	0.037511	-1.59285451
40	Tnni3	7	1.12E-05	0.010033	-1.60608144
41	Fabp3	4	3.23E-06	0.003997	-1.64030512
42	Efcab2	1	3.73E-05	0.023079	-1.65422805
43	Acss1	2	4.42E-07	0.001045	-1.66990303
44	Itgb1bp2	X	7.64E-05	0.033626	-1.67956939
45	Me3	7	0.000115	0.038906	-1.68421925
46	Prox1	1	1.07E-06	0.001854	-1.70002823
47	Chrm2	6	0.000186	0.047375	-1.7034078
48	Mov10l1	15	8.03E-05	0.033648	-1.71009343
49	Atp2a2	5	0.000139	0.043386	-1.72516799
50	Hfe2	3	0.000118	0.038906	-1.78057117
51	Asb11	X	8E-05	0.033648	-1.7942595
52	Gm14290	2	4.53E-05	0.023708	-1.79475912
53	Pde1c	6	0.000161	0.045792	-1.79584375
54	Cd300lg	11	9.07E-05	0.03518	-1.80510862
55	Gja5	3	8.83E-05	0.03518	-1.80662587
56	Pgam	11	3.44E-08	0.000201	-1.80845317
57	Ckmt2	13	2.41E-06	0.003483	-1.82776666
58	2010107G23Rik	10	0.000177	0.046484	-1.84135025
59	Sh2d4a	8	1.43E-05	0.011613	-1.84714625
60	Pm20d1	1	0.000177	0.046484	-1.88756631
61	Klhl31	9	2.81E-05	0.020241	-1.8890941
62	Ptgds	2	3.18E-06	0.003997	-1.92632837
63	Srrm4	5	0.000152	0.043809	-1.92644861
64	Pln	10	2.57E-05	0.019108	-1.93420311
65	Krt222	11	0.000142	0.043809	-1.94004721
66	Art1	7	2.88E-05	0.020241	-1.95682535
67	Yipf7	5	6.79E-06	0.007051	-1.97377105
68	Tuba8	6	0.000165	0.046164	-1.97773751
69	Slc25a34	4	3.7E-05	0.023079	-1.99457538
70	Crhr2	6	8.03E-05	0.033648	-2.05154728
71	Scn4a	11	9.74E-05	0.036688	-2.06290931
72	Hrc	7	1.92E-08	0.000201	-2.0862654
73	Fndc5	4	1.29E-06	0.002101	-2.09076165
74	Cmya5	13	4.27E-05	0.023708	-2.10598564
75	Fsd2	7	4.49E-05	0.023708	-2.12431042
76	Sypl	3	0.000182	0.046799	-2.18314416
77	Thbs4	13	0.000118	0.038906	-2.19730372
78	Car14	3	3.59E-05	0.023079	-2.20528605

79	B3galt2	1	7.21E-05	0.032855	-2.20837734
80	Rnf207	4	4.69E-05	0.023868	-2.21497743
81	2310050B05Rik	10	3.16E-06	0.003997	-2.23147493
82	Myot	18	3.87E-08	0.000201	-2.25052767
83	Bdh1	16	0.000134	0.042396	-2.32996083
84	Grip2	6	2.01E-06	0.003074	-2.36727023
85	Gm889	12	0.000107	0.03824	-2.39239764
86	Fmod	1	3.04E-05	0.02077	-2.40082309
87	Slc2a4	11	5.37E-06	0.006068	-2.41681375
88	Abcc8	7	5.75E-05	0.027147	-2.43957832
89	Acta1	8	5.26E-05	0.025321	-2.46561339
90	Lrrc4b	7	1.34E-05	0.011568	-2.62789749
91	2210407C18Rik	11	0.000171	0.046484	-2.73112264
92	Gck	11	1.78E-07	0.000662	-3.07921286
93	Dhrs7c	11	1.07E-05	0.009943	-3.11416848
94	Asb10	5	3.82E-07	0.000993	-3.53772842
95	Mylk4	13	9.12E-07	0.001692	-3.93333046
96	Kcnip2	19	2.92E-08	0.000201	-4.14478078
97	Fstl4	11	1.25E-07	0.000543	-4.4001082
98	Lrtm1	14	7.81E-07	0.001562	-4.95027441
99	Fam169a	13	6.37E-06	0.00689	-4.99909173
100	Gkn3	6	5.44E-07	0.001178	-5.86235283
101	Scn4	9	3.19E-07	0.00092	-6.97746025
102	Wnt9b	11	2.19E-07	0.000712	-7.07536561
103	Efemp1	11	3.44E-28	8.94E-24	-11.7135908

Supplementary Table 6. Full list of enriched/depleted pathways from GSEA analysis of bulk RNA-seq data of infarct zone tissue of *Efemp1*$^{-/-}$ and WT mice at day-3 post-MI (data of Supplementary Table 4), ranked by normalised enrichment score (NES) of pathways in *Efemp1*$^{-/-}$ mice relative to WT.

**Name**	**Normalised Enrichment Score (NES)**	**FWER p-value**
cytoplasmic ribosomal proteins%wikipathways_20190910%wp163%mus musculus	3.0064538	0
hallmark_tnfa_signaling_via_nfkb%msigdb_c2%hallmark_tnfa_signaling_via_nfkb	2.8969045	0
cytosolic ribosome%gocc%go:0022626	2.8716636	0
cytosolic small ribosomal subunit%gocc%go:0022627	2.7421553	0
cytosolic large ribosomal subunit%gocc%go:0022625	2.7093391	0
peptide chain elongation%reactome%r-hsa-156902.2	2.7075503	0
eukaryotic translation termination%reactome database id release 70%72764	2.6589925	0
nonsense mediated decay (nmd) independent of the exon junction complex (ejc)%reactome database id release 70%975956	2.6249723	0
selenocysteine synthesis%reactome database id release 70%2408557	2.6158545	0
selenoamino acid metabolism%reactome%r-hsa-2408522.4	2.6142175	0
viral mrna translation%reactome database id release 70%192823	2.5863633	0
eukaryotic translation elongation%reactome%r-hsa-156842.2	2.581837	0
formation of a pool of free 40s subunits%reactome%r-hsa-72689.2	2.5777383	0
small ribosomal subunit%gocc%go:0015935	2.571751	0
nonsense mediated decay (nmd) enhanced by the exon junction complex (ejc)%reactome%r-hsa-975957.1	2.5657604	0
srp-dependent cotranslational protein targeting to membrane%reactome database id release 70%1799339	2.5650725	0
nonsense-mediated decay (nmd)%reactome%r-hsa-927802.2	2.554698	0
interleukin-10 signaling%reactome database id release 70%6783783	2.5494666	0
cap-dependent translation initiation%reactome%r-hsa-72737.2	2.5484219	0
l13a-mediated translational silencing of ceruloplasmin expression%reactome%r-hsa-156827.3	2.5347686	0
ribosomal subunit%gocc%go:0044391	2.528233	0
gtp hydrolysis and joining of the 60s ribosomal subunit%reactome database id release 70%72706	2.5266173	0
eukaryotic translation initiation%reactome database id release 70%72613	2.5207305	0
rrna processing in the nucleus and cytosol%reactome%r-hsa-8868773.3	2.496606	0
structural constituent of ribosome%gomf%go:0003735	2.4792202	0
influenza viral rna transcription and replication%reactome database id release 70%168273	2.479186	0
major pathway of rrna processing in the nucleolus and cytosol%reactome database id release 70%6791226	2.476893	0
hallmark_allograft_rejection%msigdb_c2%hallmark_allograft_rejection	2.4503694	0
ribosomal small subunit biogenesis%gobp%go:0042274	2.3938162	0
influenza life cycle%reactome%r-hsa-168255.3	2.3936865	0
rrna processing%reactome%r-hsa-72312.3	2.3873622	0
cytoplasmic translation%gobp%go:0002181	2.341571	0

cell chemotaxis%gobp%go:0060326	2.3343358	0
influenza infection%reactome database id release 70%168254	2.314896	0
polysomal ribosome%gocc%go:0042788	2.3121495	0
leukocyte chemotaxis%gobp%go:0030595	2.3026788	0
ribosomal large subunit biogenesis%gobp%go:0042273	2.218056	0
myeloid leukocyte migration%gobp%go:0097529	2.2131307	0
cytokine activity%gomf%go:0005125	2.2036593	0
antigen processing and presentation of peptide antigen%gobp%go:0048002	2.1980789	0
ap-1 transcription factor network%pathway interaction database nci-nature curated data%ap-1 transcription factor network	2.1973357	0
rrna metabolic process%gobp%go:0016072	2.1967924	0
formation of the ternary complex, and subsequently, the 43s complex%reactome%r-hsa-72695.2	2.195465	0.001
large ribosomal subunit%gocc%go:0015934	2.1915112	0.001
regulation of expression of slits and robos%reactome%r-hsa-9010553.1	2.1866887	0.001
mhc protein complex%gocc%go:0042611	2.1865885	0.001
translation initiation complex formation%reactome%r-hsa-72649.3	2.185553	0.001
rrna processing%gobp%go:0006364	2.176273	0.002
activation of the mrna upon binding of the cap-binding complex and eifs, and subsequent binding to 43s%reactome database id release 70%72662	2.1704297	0.004
interleukin-4 and interleukin-13 signaling%reactome database id release 70%6785807	2.1662052	0.004
antigen processing and presentation%gobp%go:0019882	2.1508958	0.006
cytokines and inflammatory response%wikipathways_20190910%wp222%mus musculus	2.1351032	0.011
preribosome%gocc%go:0030684	2.1289802	0.014
rrna modification in the nucleus and cytosol%reactome database id release 70%6790901	2.1229413	0.014
ribosomal small subunit assembly%gobp%go:0000028	2.1217468	0.014
pid_ap1_pathway%msigdb_c2%pid_ap1_pathway	2.1215506	0.014
leukocyte migration%gobp%go:0050900	2.1188793	0.014
polysome%gocc%go:0005844	2.1105597	0.017
mrna splicing%reactome database id release 70%72172	2.1099062	0.017
ribosomal scanning and start codon recognition%reactome%r-hsa-72702.3	2.1094303	0.017
positive regulation of hemopoiesis%gobp%go:1903708	2.1074545	0.017
antigen processing and presentation of exogenous peptide antigen%gobp%go:0002478	2.1074343	0.017
hallmark_inflammatory_response%msigdb_c2%hallmark_inflammatory_response	2.099924	0.02
positive regulation of leukocyte differentiation%gobp%go:1902107	2.099528	0.02
spliceosomal complex%gocc%go:0005681	2.096131	0.026
pid_fra_pathway%msigdb_c2%pid_fra_pathway	2.0961044	0.026
il12-mediated signaling events%pathway interaction database nci-nature curated data%il12-mediated signaling events	2.0955372	0.026
antigen processing and presentation of peptide antigen via mhc class ii%gobp%go:0002495	2.094126	0.026
neutrophil chemotaxis%gobp%go:0030593	2.0798929	0.035
granulocyte chemotaxis%gobp%go:0071621	2.0740495	0.04

antimicrobial humoral response%gobp%go:0019730	2.0694537	0.042
antigen processing and presentation of peptide or polysaccharide antigen via mhc class ii%gobp%go:0002504	2.0685253	0.044
defense response to bacterium%gobp%go:0042742	2.067715	0.044
pre-mrna splicing%reactome%r-hsa-72163.2	2.0633993	0.047
cell-cell contact zone%gocc%go:0044291	-2.1107602	0.045
actin-mediated cell contraction%gobp%go:0070252	-2.1123347	0.043
nad binding%gomf%go:0051287	-2.1133156	0.042
cardiac conduction%gobp%go:0061337	-2.114521	0.04
atp synthesis coupled electron transport%gobp%go:0042773	-2.1174624	0.038
regulation of transporter activity%gobp%go:0032409	-2.118235	0.035
ribonucleotide biosynthetic process%gobp%go:0009260	-2.1212347	0.033
cardiac muscle tissue morphogenesis%gobp%go:0055008	-2.1226223	0.032
tricarboxylic acid cycle%gobp%go:0006099	-2.1259358	0.028
mitochondrial atp synthesis coupled electron transport%gobp%go:0042775	-2.127881	0.026
nucleoside bisphosphate metabolic process%gobp%go:0033865	-2.1302009	0.025
purine ribonucleotide biosynthetic process%gobp%go:0009152	-2.1305766	0.025
regulation of transmembrane transporter activity%gobp%go:0022898	-2.1317756	0.025
actin filament-based movement%gobp%go:0030048	-2.1340425	0.024
monovalent inorganic cation transport%gobp%go:0015672	-2.1375813	0.021
response to activity%gobp%go:0014823	-2.1405084	0.02
fatty acid beta oxidation%wikipathways_20190910%wp1269%mus musculus	-2.1407804	0.02
nucleoside monophosphate metabolic process%gobp%go:0009123	-2.141951	0.02
mitochondrial fatty acid beta-oxidation%reactome%r-hsa-77289.5	-2.1460838	0.017
structural constituent of muscle%gomf%go:0008307	-2.1474595	0.017
citrate metabolic process%gobp%go:0006101	-2.1476412	0.017
nucleoside triphosphate metabolic process%gobp%go:0009141	-2.152134	0.015
positive regulation of cation channel activity%gobp%go:2001259	-2.1560974	0.014
ribonucleoside bisphosphate metabolic process%gobp%go:0033875	-2.1667707	0.009
muscle hypertrophy%gobp%go:0014896	-2.166836	0.009
regulation of ion transmembrane transporter activity%gobp%go:0032412	-2.1688468	0.009
positive regulation of transmembrane transport%gobp%go:0034764	-2.1693652	0.008
lipid oxidation%gobp%go:0034440	-2.1718483	0.008
cell-cell signaling involved in cardiac conduction%gobp%go:0086019	-2.1789901	0.006
electron transport chain%wikipathways_20190910%wp295%mus musculus	-2.1851583	0.006
ion homeostasis%reactome database id release 70%5578775	-2.1859317	0.006
purine nucleoside bisphosphate metabolic process%gobp%go:0034032	-2.1868644	0.006
hallmark_adipogenesis%msigdb_c2%hallmark_adipogenesis	-2.189056	0.006
ribonucleoside triphosphate metabolic process%gobp%go:0009199	-2.1895318	0.006
m band%gocc%go:0031430	-2.1902695	0.006
fatty acid oxidation%gobp%go:0019395	-2.1916296	0.006
regulation of cardiac muscle cell contraction%gobp%go:0086004	-2.1952913	0.006

oxidative phosphorylation%gobp%go:0006119	-2.1969824	0.006
fatty acid beta-oxidation%gobp%go:0006635	-2.1992114	0.006
mitochondrial membrane part%gocc%go:0044455	-2.199397	0.006
striated muscle contraction%reactome%r-hsa-390522.1	-2.204785	0.006
regulation of muscle contraction%gobp%go:0006937	-2.2052815	0.005
ribonucleoside monophosphate metabolic process%gobp%go:0009161	-2.2074702	0.005
cardiac conduction%reactome%r-hsa-5576891.2	-2.2141383	0.004
purine ribonucleoside monophosphate metabolic process%gobp%go:0009167	-2.2164743	0.004
thioester metabolic process%gobp%go:0035383	-2.222253	0.004
regulation of cardiac muscle cell action potential%gobp%go:0098901	-2.222693	0.003
ion channel binding%gomf%go:0044325	-2.2235177	0.003
a band%gocc%go:0031672	-2.224574	0.003
regulation of actin filament-based movement%gobp%go:1903115	-2.2253802	0.003
striated muscle thin filament%gocc%go:0005865	-2.228663	0.002
purine nucleoside triphosphate metabolic process%gobp%go:0009144	-2.2287037	0.002
acyl-coa metabolic process%gobp%go:0006637	-2.2330487	0.001
positive regulation of ion transmembrane transport%gobp%go:0034767	-2.2335637	0.001
oxidoreductase complex%gocc%go:1990204	-2.234014	0.001
regulation of heart rate%gobp%go:0002027	-2.236217	0
positive regulation of cation transmembrane transport%gobp%go:1904064	-2.2378032	0
positive regulation of ion transmembrane transporter activity%gobp%go:0032414	-2.2416058	0
striated muscle hypertrophy%gobp%go:0014897	-2.242228	0
purine ribonucleoside triphosphate metabolic process%gobp%go:0009205	-2.2429605	0
cardiac muscle hypertrophy%gobp%go:0003300	-2.2436388	0
purine nucleoside monophosphate metabolic process%gobp%go:0009126	-2.2440038	0
positive regulation of transporter activity%gobp%go:0032411	-2.2579944	0
cardiocyte differentiation%gobp%go:0035051	-2.2588487	0
intercalated disc%gocc%go:0014704	-2.259433	0
aerobic respiration%gobp%go:0009060	-2.259468	0
citric acid cycle (tca cycle)%reactome%r-hsa-71403.2	-2.2619166	0
hallmark_fatty_acid_metabolism%msigdb_c2%hallmark_fatty_acid_metabolism	-2.2677824	0
regulation of blood circulation%gobp%go:1903522	-2.2688324	0
atp metabolic process%gobp%go:0046034	-2.2695143	0
myofilament%gocc%go:0036379	-2.2783234	0
muscle fiber development%gobp%go:0048747	-2.278475	0
respiratory electron transport%reactome database id release 70%611105	-2.2939274	0
regulation of cardiac muscle contraction%gobp%go:0055117	-2.296253	0
respiratory electron transport chain%gobp%go:0022904	-2.2968402	0
t-tubule%gocc%go:0030315	-2.3000574	0
regulation of striated muscle contraction%gobp%go:0006942	-2.3049545	0
muscle contraction%reactome database id release 70%397014	-2.306137	0

respiratory electron transport, atp synthesis by chemiosmotic coupling, and heat production by uncoupling proteins.%reactome%r-hsa-163200.1	-2.3086336	0
cardiac muscle tissue development%gobp%go:0048738	-2.31471	0
electron transport chain%gobp%go:0022900	-2.3150637	0
tca cycle%wikipathways_20190910%wp434%mus musculus	-2.3211567	0
muscle contraction%gobp%go:0006936	-2.3331597	0
sarcomere organization%gobp%go:0045214	-2.33869	0
striated muscle contraction%wikipathways_20190910%wp216%mus musculus	-2.3416383	0
muscle system process%gobp%go:0003012	-2.351891	0
sarcolemma%gocc%go:0042383	-2.3573024	0
cell communication involved in cardiac conduction%gobp%go:0086065	-2.3617103	0
generation of precursor metabolites and energy%gobp%go:0006091	-2.3798501	0
cellular component assembly involved in morphogenesis%gobp%go:0010927	-2.391215	0
heart process%gobp%go:0003015	-2.3988144	0
cardiac muscle contraction%gobp%go:0060048	-2.399019	0
regulation of heart contraction%gobp%go:0008016	-2.41405	0
striated muscle contraction%gobp%go:0006941	-2.4167225	0
cardiac muscle cell differentiation%gobp%go:0055007	-2.4219186	0
sarcoplasm%gocc%go:0016528	-2.4227865	0
striated muscle cell differentiation%gobp%go:0051146	-2.4245431	0
myofibril assembly%gobp%go:0030239	-2.4257855	0
heart contraction%gobp%go:0060047	-2.4326525	0
cardiac cell development%gobp%go:0055006	-2.4419627	0
cellular respiration%gobp%go:0045333	-2.4455566	0
pyruvate metabolism and citric acid (tca) cycle%reactome database id release 70%71406	-2.4493217	0
cardiac muscle cell development%gobp%go:0055013	-2.4574206	0
actomyosin structure organization%gobp%go:0031032	-2.4587104	0
sarcoplasmic reticulum%gocc%go:0016529	-2.4692338	0
hallmark_myogenesis%msigdb_c2%hallmark_myogenesis	-2.4713929	0
muscle cell development%gobp%go:0055001	-2.472081	0
energy derivation by oxidation of organic compounds%gobp%go:0015980	-2.4812691	0
hallmark_oxidative_phosphorylation%msigdb_c2%hallmark_oxidative_phosphorylation	-2.4841175	0
striated muscle cell development%gobp%go:0055002	-2.5667367	0
z disc%gocc%go:0030018	-2.572716	0
the citric acid (tca) cycle and respiratory electron transport%reactome%r-hsa-1428517.1	-2.576848	0
contractile fiber part%gocc%go:0044449	-2.6347687	0
contractile fiber%gocc%go:0043292	-2.644069	0
i band%gocc%go:0031674	-2.6624746	0
sarcomere%gocc%go:0030017	-2.687026	0
myofibril%gocc%go:0030016	-2.6920128	0

Supplementary Table 7. Cytoscape search function was used to pull out and highlight the core genes encompassing pathways of interest which were either enriched/up or depleted/down in *Efemp1*$^{-/-}$ mice relative to WT (from Supplementary Table 5 data). Pathways are listed vertically in the first column, and the top 10 core genes involved in those pathways are listed by rank left to right (horizontally, columns 2-11). Data are separated by enriched/up (presented first) and depleted/down in *Efemp1*$^{-/-}$ mice.

**Up in Efemp1-/-**	**1**	**2**	**3**	**4**	**5**	**6**	**7**	**8**	**9**	**10**
AP-1 Transcription Factor Network	FOSB	MMP9	ATF3	IL6	H2-Q10	JUN	JUNB	FOSL1	MT2	FOSL2
ATF2 Transcription Factor Network	ATF3	IL6	JUN	PDGFRA	HES1	JUNB	DUSP5	SOCS3	JDP2	FOS
Cellular Response to Cadmium Ion	MMP9	JUN	MT2	FOS	GSN	MT1	EGFR	HSF1	SUMO1	HMOX1
Cytokine Activity	CCL3	IL6	IL1B	LIF	IL1RN	CXCL1	CXCL3	CXCL2	OSM	INHBA
Cytokine Receptor Binding	CCL3	NGF	IL6	IL1B	LIF	IL1RN	CXCL1	CXCL3	SMAD3	PGF
Cytokine Inflammatory Response	IL6	IL1B	H2-EB1	CXCL1	CXCL3	IL11	CSF1	TNF	CD4	TGFB1
Hallmark Allograft Rejection	MMP9	IL6	IL1B	H2-Q10	LIF	H2-OA	H2-AA	LTB	NCF4	INHBA
Hallmark Inflammatory Response	HAS2	TNFAIP 6	IL6	IL1B	ADM	LIF	KCNJ2	CSF3R	OSM	SPHK1
IL4 IL13 Signaling	MMP9	PTGS2	MMP3	IL6	IL1B	LIF	OSM	JUNB	CCL11	FGF2
IL12 Signaling Mediated by Stat4	H2-EB1	JUN	CD80	CD86	FOS	CD4	IL18RA P	TGFB1	IL2RA	
Lung Fibrosis	CCL3	MMP9	IL6	IL1B	CXCL2	MT2	CCL11	FGF2	SMAD7	CCL5
Myeloid Leukocyte Activation	JUN	FCGR2B	PRKCD	LBP	FPR2	NR4A3	CCL5	FCGR4	TNF	JMJD6
NABA Secreted Factors	CCL3	S100A8	S100A9	NGF	IL6	IL1B	IGF2	LIF	IL1RN	CXCL14
Osteopontin Mediated Events	MMP9	JUN	NFKBIA	FOS	MAP3K1 4	GSN	ITGB3	PLAU	PTK2B	
PID AP-1 Pathway	FOSB	MMP9	ATF3	IL6	H2-Q10	JUN	JUNB	FOSL1	MT2	FOSL2
PID ATF2 Pathway	ATF3	IL6	JUN	PDGFRA	HES1	JUNB	DUSP5	SOCS3	JDP2	FOS

PID AVB3 OPN Pathway	MMP9	JUN	NFKBIA	FOS	MAP3K1 4	GSN	ITGB3	PLAU	PTK2B	
PID FRA Pathway	MMP9	IL6	MGP	LIF	JUN	JUNB	FOSL1	PLAUR	DCN	FOSL2
PID IL12 Stat4 Pathway	H2-EB1	JUN	CD80	CD86	FOS	CD4	IL18RA P	TGFB1	IL2RA	
Pos Reg Acute Inflammatory Pathway	PTGS2	IL6	IL1B	FFAR2	CCL5	TNF	MIF	TNFSF11	FCGR3	ALOX5AP
Pos Reg Fibroblast Proliferation	JUN	PDGFR A	SPHK1	TGIF1	CD74	FOSL2	EREG	CDKN1A	DDR2	FBLN1
Reg Acute Inflammatory Pathway	PTGS2	IL6	IL1B	SERPING 1	CFH	CD55	FFAR2	FCGR2B	NLRP3	A2M
Reg Cell Killing	H2-Q10	IGF2	CXCL1	CFH	CD55	H2-Q7	CLEC2 D	PGLYRP 1	ARRB2	KLRB1B
Reg Cysteine type Endopeptidase Apoptosis Signaling	MMP9	SMAD3	PLAUR	GSN	BAX	JAK2	HTRA2	FAS	TFAP4	
Reg Fibroblast Proliferation	MMP9	NUPR1	PMAIP1	JUN	PDGFRA	SPHK1	TGIF1	CD74	FOSL2	EREG
Reg Leukocyte Mediated Immunity	IL6	IL1B	H2-Q10	IGF2	CXCL1	CD55	FGR	H2-Q7	CLEC2D	FCGR2B
Reg Leukocyte Proliferation	LRRC32	IL6	IL1B	ARG2	IGF2	H2-AA	H2-AB1	HES1	CCDC88 B	CD80
Reg Lymphocyte Proliferation	LRRC32	IL6	IL1B	ARG2	IGF2	H2-AA	H2-AB1	HES1	CCDC88 B	CD80
Reg Mononuclear Cell Proliferation	LRRC32	IL6	IL1B	ARG2	IGF2	H2-AA	H2-AB1	HES1	CCDC88 B	CD80
Spinal Cord Injury	MMP9	NR4A1	IL6	IL1B	ZFP36	CXCL1	LTB	CXCL2	CHST11	KLK8
Validated Transcriptional Targets of AP-1 Family FRA1 FRA2	MMP9	IL6	MGP	LIF	JUN	JUNB	FOSL1	PLAUR	DCN	FOSL2

**Down in Efemp1-/-**	**1**	**2**	**3**	**4**	**5**	**6**	**7**	**8**	**9**	**10**
Pos Reg Cation Ion Transmembrane Transport	KCNIP2	KCNJ11	CACNA1C	ABCC8	TRDN	GBAS	ADRB1	CHCHD10	GSTM7	CACNA2D1
Pos Reg Ion Transmembrane Transport	KCNIP2	KCNJ11	CACNA1C	ABCC8	TRDN	GBAS	ADRB1	CHCHD10	GSTM7	CACNA2D1
Pos Reg Transmembrane Transport	KCNIP2	KCNJ11	CACNA1C	ABCC8	TRDN	GBAS	ERBB4	ADRB1	CHCHD10	GSTM7
Reg Calcium Ion Transmembrane Transport	DHRS7C	CAV3	CACNA1C	TRDN	GBAS	TMEM38A	ADRB1	CASQ2	GSTM7	CACNA2D1
Reg Cation Channel Activity	AMIGO1	STOM	GNB5	KCNE1	DYSF	HCN1	NPPA	HOMER1	PRKG1	GSG1L
Reg Ion Transmembrane Transporter Activity	AHNAK	WNK1	HECW2	AMIGO1	STOM	GNB5	KCNE1	DYSF	SCN3B	HSPA2
Reg Release Sequestered Calcium into Cytosol	DHRS7C	TRDN	TMEM38A	CASQ2	GSTM7	JPH2	APLNR	RYR2	ANK2	AKAP6
Reg Transmembrane Transporter Activity	AHNAK	WNK1	HECW2	AMIGO1	STOM	GNB5	KCNE1	DYSF	SCN3B	HSPA2
Reg Transporter Activity	AHNAK	WNK1	HECW2	AMIGO1	STOM	GNB5	KCNE1	DYSF	SCN3B	HSPA2

References

1 Farbehi, N. *et al.* Single-cell expression profiling reveals dynamic flux of cardiac stromal, vascular and immune cells in health and injury. *Elife* **8**, doi:10.7554/eLife.43882 (2019).

2 Vidal, R. *et al.* Transcriptional heterogeneity of fibroblasts is a hallmark of the aging heart. *JCI insight* **4**, doi:10.1172/jci.insight.131092 (2019).

3 Soliman, H. *et al.* Pathogenic potential of hic1-expressing cardiac stromal progenitors. *Cell Stem Cell* **26**, 205-220 e208, doi:10.1016/j.stem.2019.12.008 (2020).

4 Kim, J. O. *et al.* A novel system-level approach using rna-sequencing data identifies mir-30-5p and mir-142a-5p as key regulators of apoptosis in myocardial infarction. *Sci Rep* **8**, 14638, doi:10.1038/s41598-018-33020-x (2018).

5 Stuart, T. *et al.* Comprehensive integration of single-cell data. *Cell* **177**, 1888-1902 e1821, doi:10.1016/j.cell.2019.05.031 (2019).

6 Dobin, A. *et al.* Star: Ultrafast universal rna-seq aligner. *Bioinformatics* **29**, 15-21, doi:10.1093/bioinformatics/bts635 (2013).

7 Bolger, A. M., Lohse, M. & Usadel, B. Trimmomatic: A flexible trimmer for illumina sequence data. *Bioinformatics* **30**, 2114-2120, doi:10.1093/bioinformatics/btu170 (2014).

8 Love, M. I., Huber, W. & Anders, S. Moderated estimation of fold change and dispersion for rna-seq data with deseq2. *Genome Biol* **15**, 550, doi:10.1186/s13059-014-0550-8 (2014).

9 Thavapalachandran, S. *et al.* Platelet-derived growth factor-ab improves scar mechanics and vascularity after myocardial infarction. *Science translational medicine* **12**, doi:10.1126/scitranslmed.aay2140 (2020).

10 Farbehi, N. *et al.* Single-cell expression profiling reveals dynamic flux of cardiac stromal, vascular and immune cells in health and injury. *eLife* **e43882**, doi:10.7554/eLife.43882 (2019).

11 Gao, X. M., Xu, Q., Kiriazis, H., Dart, A. M. & Du, X. J. Mouse model of post-infarct ventricular rupture: Time course, strain- and gender-dependency, tensile strength, and histopathology. *Cardiovasc Res* **65**, 469-477, doi:10.1016/j.cardiores.2004.10.014 (2005).

12 Cao, J. *et al.* Estrogen induces cardioprotection in male c57bl/6j mice after acute myocardial infarction via decreased activity of matrix metalloproteinase-9 and increased akt-bcl-2 anti-apoptotic signaling. *Int J Mol Med* **28**, 231-237, doi:10.3892/ijmm.2011.681 (2011).

13 Fang, L. *et al.* Differences in inflammation, mmp activation and collagen damage account for gender difference in murine cardiac rupture following myocardial infarction. *J Mol Cell Cardiol* **43**, 535-544, doi:10.1016/j.yjmcc.2007.06.011 (2007).

14 Cavasin, M. A., Tao, Z., Menon, S. & Yang, X. P. Gender differences in cardiac function during early remodeling after acute myocardial infarction in mice. *Life Sci* **75**, 2181-2192, doi:10.1016/j.lfs.2004.04.024 (2004).

15 Villalba-Orero, M. *et al.* Lung ultrasound as a translational approach for non-invasive assessment of heart failure with reduced or preserved ejection fraction in mice. *Cardiovasc Res* **113**, 1113-1123, doi:10.1093/cvr/cvx090 (2017).

16 Farbehi, N. *et al.* Single-cell expression profiling reveals dynamic flux of cardiac stromal, vascular and immune cells in health and injury. *eLife* **8**, e43882, doi:10.7554/eLife.43882 (2019).
